# Changes of vitamin D receptors (VDR) and MAPK activation in cytoplasmic and nuclear fractions following exposure to cigarette smoke with or without filter in rats

**DOI:** 10.1016/j.heliyon.2021.e05927

**Published:** 2021-01-30

**Authors:** Fatist Okrit, Poonchavist Chantranuwatana, Duangporn Werawatganon, Maneerat Chayanupatkul, Sompol Sanguanrungsirikul

**Affiliations:** a Department of Physiology, Faculty of Medicine, Chulalongkorn University, Bangkok, 10330, Thailand; b Department of Pathology, Faculty of Medicine, Chulalongkorn University, Bangkok, 10330, Thailand

**Keywords:** Vitamin D receptors (VDR) distribution, Cigarette smoke exposure (CSE), Mitogen activated protein kinases (MAPKs)

## Abstract

Cigarette smoke (CS) is a major cause of obstructive lung disease which is associated with significant disability and mortality. Vitamin D receptor (VDR) together with, mitogen activated protein kinases (MAPKs; ERK, JNK and p38) are the cellular transmission signals that mechanistically respond to CS and are recently found to have a role in lung pathogenesis. There are a few *in vitro* studies on subcellular VDR distribution involved MAPK but *in vivo* effects of cigarette smoke exposure with and without filter on this complex remain unclear. This study investigated subcellular VDR distribution and MAPK expression at early stages of both types of cigarette smoke exposure (CSE) in a rat model. Male Wistar rats were randomly divided into no-filter, filter and control groups. After 7 and 14 days of CSE, lung tissues were obtained to determine histopathology and protein expression. Cytoplasmic and nuclear VDR distribution significantly decreased on both CSE groups and corresponded with immunohistochemistry detection. The ratio of phosphorylated ERK to total ERK significantly increased in cytoplasm of both CSE on day 7. In particular, nuclear ERK MAPK significantly escalated in the filter group on day 14. In consistent with changes in intracellular markers, histopathological examination in both CSE groups showed significant increases in tracheal and peribronchiolar epithelial proliferation, alveolar macrophages and an increased trend of parenchymal infiltration. In summary, the evidence of lung injuries along with VDR depletion and MAPK activation observed in both CSE types indicated that there was no benefit of using cigarette filter to prevent protein damage or protect cells against cigarette smoke exposure in this model.

## 1 Introduction

Cigarette smoke is the major risk factor of chronic obstructive pulmonary disease (COPD) which is the third leading cause of mortality globally [1]. Cigarette smoke is composed of more than 7,000 substances [2] which are associated with a wide variety of human pathologies, such as airway inflammation, COPD, atherosclerosis and age-related disorders [3].

Mitogen activated protein kinases (MAPKs) are intracellular molecules consisting of three subtypes, which are extracellular regulated kinase (ERK), stress activated protein kinase or c-Jun N-terminal kinase (JNK) and p38. All of these are involved in various cellular activities including cell proliferation, differentiation and cell survival [4]. Each cascade operates through the phosphorylation process and the stimulation of sequential kinases to regulate protein targets. These targets are located both in the cytoplasm and in the nucleus [5, 6, 7].

Vitamin D receptor (VDR) can be found both in the cytosol and in the nucleus and is associated with many biological actions through the interaction with active vitamin D (1,25 dihydroxy vitamin D). VDR not only has a crucial role in classical calcium and phosphate homeostasis but also has non-classical functions in regulating cell differentiation, inflammation and apoptosis in human diseases [8, 9]. According to the knockout mice model by Isaac K. Sundar et al., VDR deficient condition in mouse lung could lead to inflammatory cell infiltration, nuclear factor kappa B (NF-kB) and matrix metalloproteinases (MMPs) up-regulation that were responsible for early emphysema pathogenesis [10]. In human alveolar basal epithelial cells (A549 cells), VDR mRNA levels decreased while inflammatory cytokine mRNA levels, such as macrophage inflammatory protein 1a (MIP-1a), interferon γ-induced protein 10 (IP-10) and MMP-12, increased under cigarette smoke exposure (CSE). Hence, these findings indicated that VDR might act as anti-inflammation in COPD [11].

Uh ST et al. also reported that CSE reduced the level of cytosolic VDR distribution in human lung epithelial cells (A549 cells) with up-regulation of ERK signaling but no changes in JNK and p38 [12]. A recent phosphoproteomic study showed differential phosphorylation of multiple kinases in response to chronic cigarette smoke exposure [13]. The aforementioned studies used *in vitro* methods to investigate the effects of acute and chronic cigarette smoke exposure on VDR and MAPK cascades which provided some detailed information. However, we lack an *in vivo* study to confirm that similar biological mechanisms happen in a setting that more resembles a human disease. Therefore, in this study, we used cytoplasmic and nuclear fractions of VDR to describe their activities and alterations at different stages after cigarette smoke exposure. Furthermore, investigating the dynamics between subcellular MAPK cascades and VDR distribution would provide a better insight into physiological responses and cellular pathobiology after CSE. In addition, the effects of cigarette smoke filter on the distribution of MAPK cascades and VDR proteins in cytoplasm and nucleus of rat lung tissues in an acute phase of cigarette smoke exposure have never been explored. The aim of this study was to investigate the effects of cigarette smoke exposure with or without filter on cytoplasmic and nuclear translocation of VDR, ERK, JNK, and p38 pathways *in vivo*.

## 2 Materials and methods

### 2.1 Animal preparation

Male Wistar rats (age 6–8 weeks, body weight 210–250 g) were purchased from the National Laboratory Animal Center, Salaya Campus, Mahidol University, Nakhon Pathom, Thailand. Experimental procedures were conducted in accordance with Ethical Principles and Guidelines for the Use of Animals by National Research Council of Thailand, and approved by the Institutional Animal Care & Use Committee (IACUC), Faculty of Medicine, Chulalongkorn University (IRB approval number: 9/59). All animals were housed at 25 °C with 12:12 h light-dark cycle and allowed access to standard diet and water ad libitum.

### 2.2 Smoke exposure procedures

**Figure 1:**
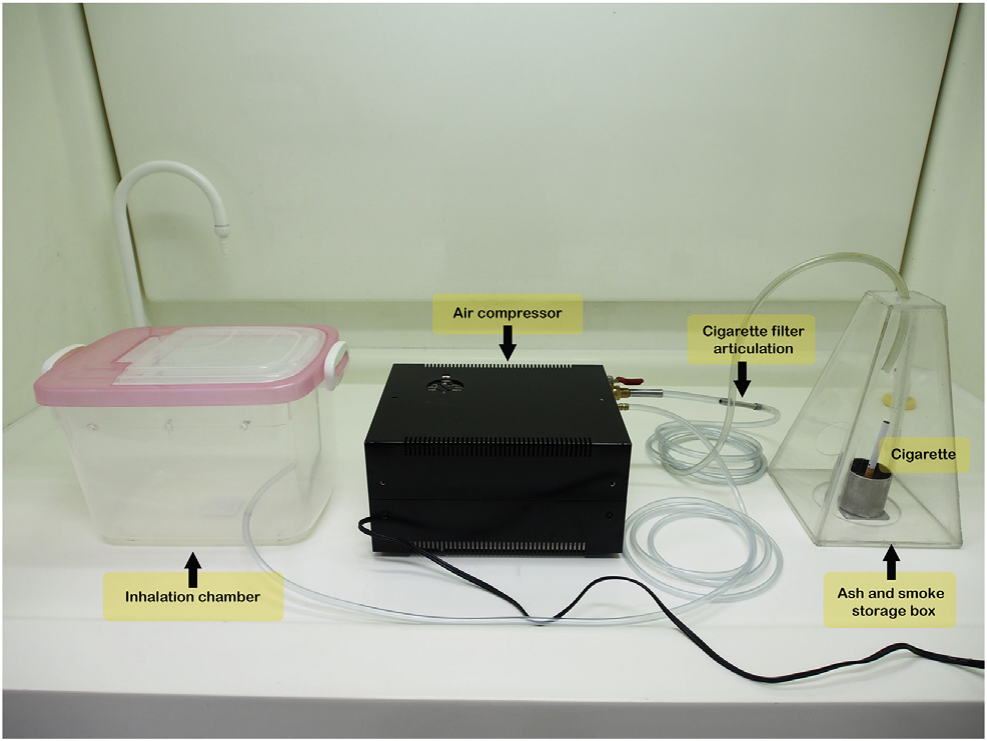
Components of cigarette smoke exposure system. The cigarette smoke exposure system was composed of cigarette, ash and smoke storage box, cigarette filter articulation, air compressor and inhalation chamber (Right to left).

Thirty male Wistar rats were randomly divided into three major groups (n = 10 per group); 1) control group, 2) CS with no-filter, and 3) CS with filter. Rats in each group were sub-divided into 7 and 14 days of exposure groups (n = 5 in each subgroup). The exposure system was composed of five components; air compressor, cigarette filter articulation, the inhalation chamber, cigarette container, ash and smoke storage box (Figure 1) as previously described [14].

For the cigarette smoke with filter group, rat was placed in the inhalation chamber one by one, and then the cigarette was lit and covered with commercial cigarette filter connected with the cigarette filter articulation. The air compressor inhaled smoke from the storage box and squirted to the animal in the chamber. In the cigarette smoke with no-filter group, the filter was not inserted at the articulation and the smoke was directly exposed to the animal. All rats in cigarette smoke groups were exposed to the smoke inhalation for the total of 90 min. Each rat was placed in the inhalation chamber twice a day at 10.00 am and 2.00 pm using 3 cigarettes per each period for the total of 7 or 14 days depending on the group. This smoke exposure protocol was to imitate smokers’ behavior. For the control group, rats were placed in the inhalation chamber and exposed to room air instead of cigarette smoke with the same exposure protocol as the experiment groups. All rats were euthanized at 24 h after the last experiment.

### 2.3 Lung tissue preparation and histopathological examination

After 24 h from the last exposure, rats were euthanized by overdose intraperitoneal sodium pentobarbital injection and both lungs were collected. The right lung was frozen and kept in liquid nitrogen at -80 °C until analysis. The left lung was instilled and stored in 10% neutral buffered formalin. Then, a 4μm section of left basal lung was cut, dehydrated, embedded in paraffin block and stained with hematoxylin and eosin for the evaluation of tracheal epithelial cell changes, peribronchiolar proliferation, lung parenchymal infiltration and alveolar macrophage count. All fields in each section were examined by an experienced pathologist who was blinded to the experiment groups. All parameters were graded semi-quantitatively using the modified criteria described by Dogan et al. [15]. Tracheal epithelial cell change was graded as score 0 = no change, 1 = focal squamous cell metaplasia, 2 = squamous cell metaplasia with hyperplasia,3 = squamous cell metaplasia with hyperplasia and acute inflammation and 4 = squamous cell metaplasia with hyperplasia and more diffuse inflammation. Peribronchiolar epithelial cell proliferation was graded as score 0 = normal two-layered epithelium, 1 = squamous dysplasia (injury to 25%), 2 = mild dysplasia (injury to 50%), 3 = moderate dysplasia (injury to 75%) and 4 = severe dysplasia (more than 75%). Lung parenchymal infiltration was graded as 0 = absence of injury, 1 = injury to 25% of the field, 2 = injury to 50% of the field, 3 = injury to 75% of the field and 4 = more than 75% of the field (diffuse injury). Alveolar macrophage count was graded as 0 = absence of alveolar macrophage cell, 1 = 1–4 cells, 2 = 5–9 cells, 3 = ≥10 cells and 4 = abundant cells (suppl. Table 1).

**Table 1:** Histopathological scores of rat lungs following exposure to cigarette smoke.

	Day 7			Day 14		
	Control	Non-filter	Filter	Control	Non-filter	Filter
Tracheal epithelial changes	0.00 ± 0.00	1.40 ± 0.51	1.00 ± 0.31	0.00 ± 0.00	2.20 ± 0.49∗∗	1.60 ± 0.40∗∗
Peribronchiolar epithelial proliferation	0.00 ± 0.00	2.40 ± 0.67∗	0.80 ± 0.80	0.00 ± 0.00	2.60 ± 0.40∗∗	1.40 ± 0.51
Lung parenchymal infiltration	0.50 ± 0.00	0.80 ± 0.12	0.90 ± 0.29	0.50 ± 0.00	1.00 ± 0.00	0.90 ± 0.18
Alveolar macrophages count	0.52 ± 0.08	1.60 ± 0.16∗	1.48 ± 0.31∗	0.64 ± 0.11	1.64 ± 0.14∗∗	1.80 ± 0.32∗∗

∗p < 0.05 when compared with the control group at day 7, ∗∗p < 0.05 when compared with the control group at day 14 (n = 5 per each group). Data were expressed as mean ± standard error of mean (SEM).

### 2.4 Immunohistochemistry for VDR expression

The immunohistochemistry was performed to investigate the VDR localization in different conditions of cigarette smoke exposure in rats’ lungs. Most of the reagents were purchased from DAKO envision system (Dako, CA, USA). Tissue sections from paraffin block were sliced at 3 μm thickness, then deparaffinized, rehydrated and retrieved antigen with citrate buffer at pH 6.0 in microwave for 13 min. Slides were incubated with 3%H_2_O_2_ to block endogenous peroxidase and washed with wash buffer for 5 min/step. Subsequently, slides were coated with antibody diluent for 10 min to block non-specific binding and incubated with a polyclonal VDR primary antibody (dilution 1:300, NOVUS, Littleton, USA) at 4 °C overnight. Sliced were then incubated with secondary antibody (Dako, CA, USA) for 30 min and diaminobenzidine (DAB) was used for a color development. Sections were counterstained with hematoxylin. Under light microscope, positive cells were counted in five random fields at 40x magnification and expressed as VDR-positive alveolar type II cells per lung tissues area (μm2) [16].

### 2.5 Cytoplasmic and nuclear protein extraction from lung tissues

Nuclear and cytoplasmic protein extractions were performed using NE-PER nuclear and cytoplasmic extraction reagent kit (Thermo Scientific, IL, USA) following the guideline from the manufacturer. The frozen rat-lung tissue (20–100 mg) was homogenized using ice-cold cytoplasmic extraction reagent I, II (CER I, II) containing Halt protease and phosphatase inhibitor cocktail (Thermo Scientific, IL, USA) and centrifuged at 16,000×g at 4 °C for 5 min. Lung lysate was prepared separately for each rat. The supernatant was transferred (cytoplasmic extraction) to a clean tube and kept at -80 °C until used. The cell pellet was suspended and vortexed for 15 s every 10 min for 60 min on ice-cold nuclear extraction reagent (NER). The sample was centrifuged at 16,000 g for 10 min and supernatant (nuclear extraction) was immediately transferred into a clean pre-chilled tube at -80 °C. The protein concentration of both cytoplasmic and nuclear protein extractions was determined by Pierce BCA assay kit (Thermo Scientific, IL, USA).

### 2.6 Western blot analysis

Fifty micrograms of lung samples were separated on 10% sodium dodecyl sulfate polyacrylamide gel electrophoresis (SDS-PAGE) and blotted on 0.45μm Polyvinylidine-fluoride (PVDF) membrane (Merck, Millipore, Billerica, MA) by wet tank manner [17]. Blots were blocked with 1% bovine serum albumin (BSA) in phosphate-buffered saline (PBS) plus 0.01% Tween 20 at 4 °C overnight and incubated with primary antibody for 1 h at room temperature. Primary antibodies and dilution were as follows: VDR (1:1,000, Novus Biologicals), total ERK (1:1,000), phosphor-ERK (1:1,000), total JNK (1:1,000), phosphor-JNK (1:1,000), total p38 (1:1,000) and phosphor-p38 (1:1,000) (Cell Signaling, CA, USA). Blots were incubated with the HRP-conjugated anti-mouse IgG secondary antibody for 60 min at room temperature. β-actin (1:2,000, Cell signaling, CA, USA) and lamin B1 (1:10,000, Abcam, MA, USA) were used as the loading controls for cytoplasmic and nuclear protein, respectively. The signal was developed by ECL kit (Thermo Scientific) and visualized with ChemiDoc Touch Imaging system (Bio-rad).

### 2.7 Statistical analysis

Data were expressed as mean ± standard error of mean (SEM). One-way analysis of variance (one-way ANOVA) and Tukey's HSD *post hoc test* were used to compare continuous data among groups. Differences were considered statistically significant at p-value less than 0.05 (p < 0.05). All statistical analyses were performed using SPSS software version 22 (SPSS, Inc., Chicago, IL, USA).

## 3 Results

### 3.1 Effects of cigarette smoke exposure with filter and no-filter on lung histopathological changes and inflammation

**Figure 2:**
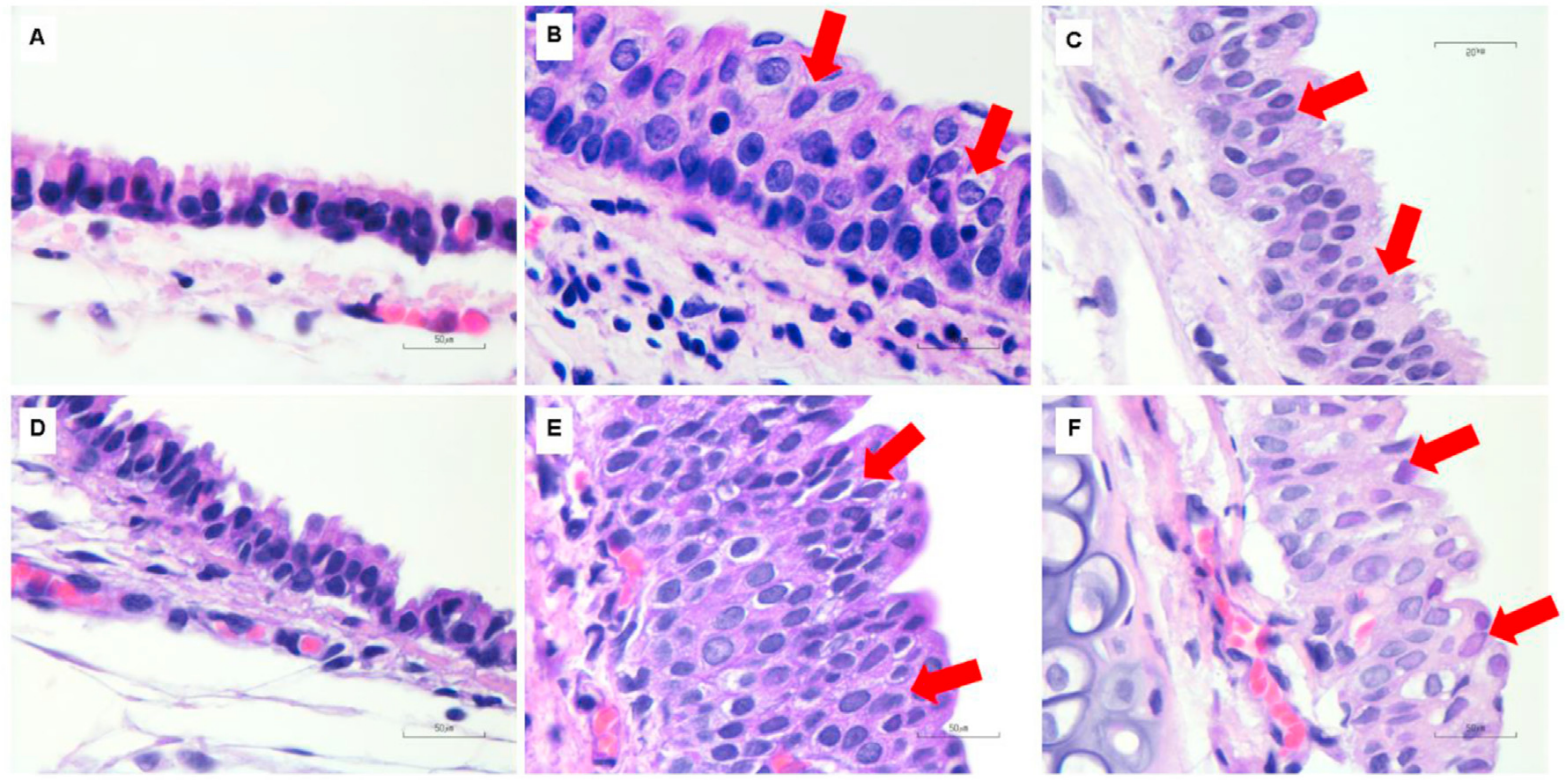
Histological features of tracheal epithelial cell changes in rat lungs using H&E staining (100X, n = 5 per group). (A,D) Tracheal epithelial cells of control rats after being exposed to room air for 7 and 14 days showed normal epithelial layers. (B,E) Non-filtered CSE groups at 7 and 14 days, and (C,F) filtered CSE groups at 7 and 14 days showed mild to moderate squamous cell metaplasia. Red arrows indicate mild to moderate squamous cell metaplasia.

**Figure 3:**
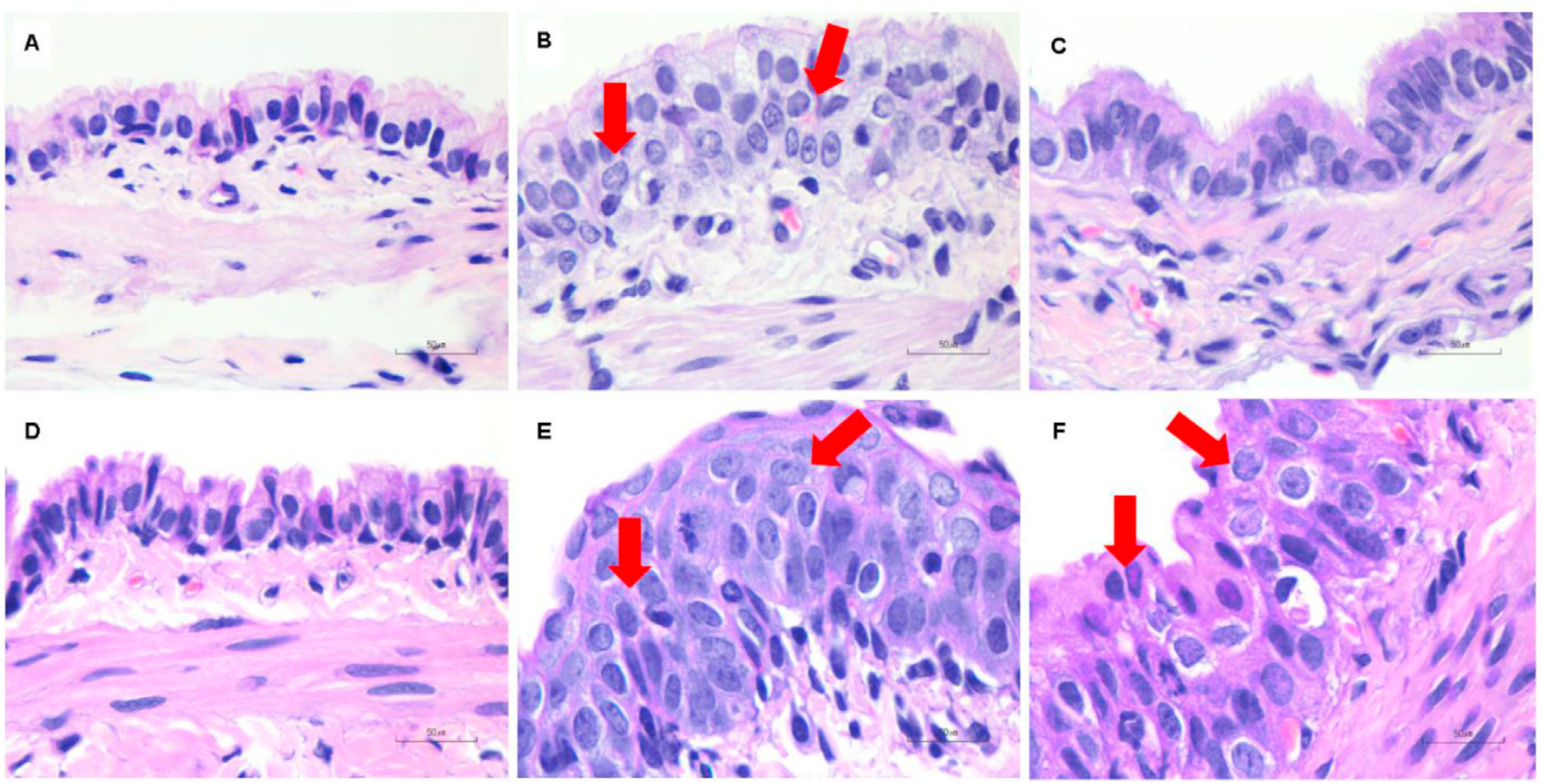
Histological features of peribronchiolar epithelial cell proliferation in rat lungs using H&E staining (100X, n = 5 per group). (A,D) Peribronchiolar epithelial cells of control rats after being exposed to room air for 7 and 14 days showed normal epithelial layers. (B,E) Non-filtered CSE groups at 7 and 14 days, and (C,F) filtered CSE groups at 7 and 14 days showed mild to moderate epithelial cell dysplasia. Red arrows indicate mild to moderate epithelial cell dysplasia.

**Figure 4:**
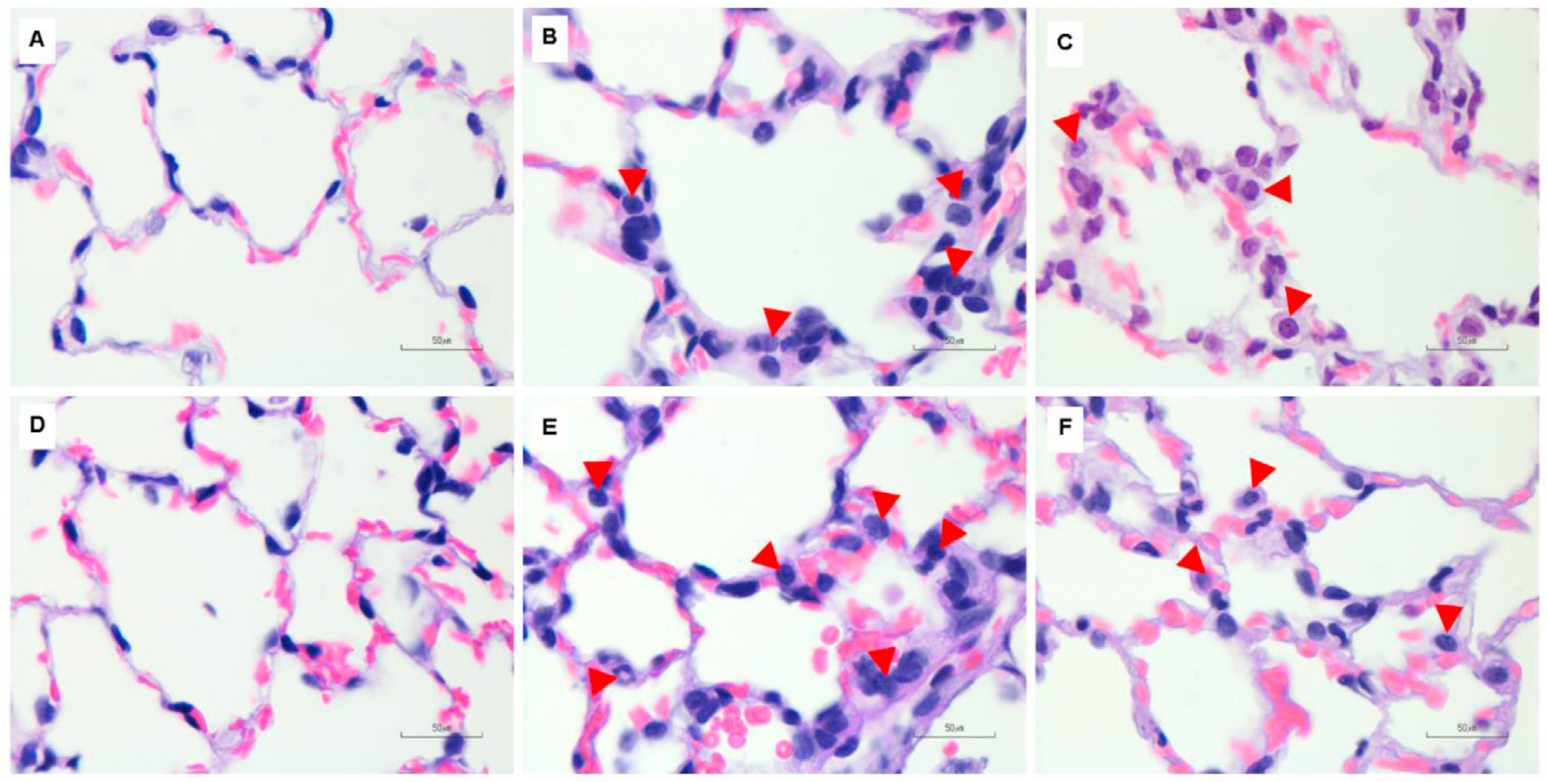
Histological features of lung parenchymal infiltration in rat lungs using H&E staining (100X, n = 5 per group). (A,D) Lung parenchymal infiltration of control rats after being exposed to room air for 7 and 14 days showed few inflammatory cells. (B,E) Non-filtered CSE groups and (C,F) filtered CSE groups at 7 and 14 days showed increased inflammatory cell infiltration. Red arrows indicate inflammatory cell infiltration in lung parenchyma.

**Figure 5:**
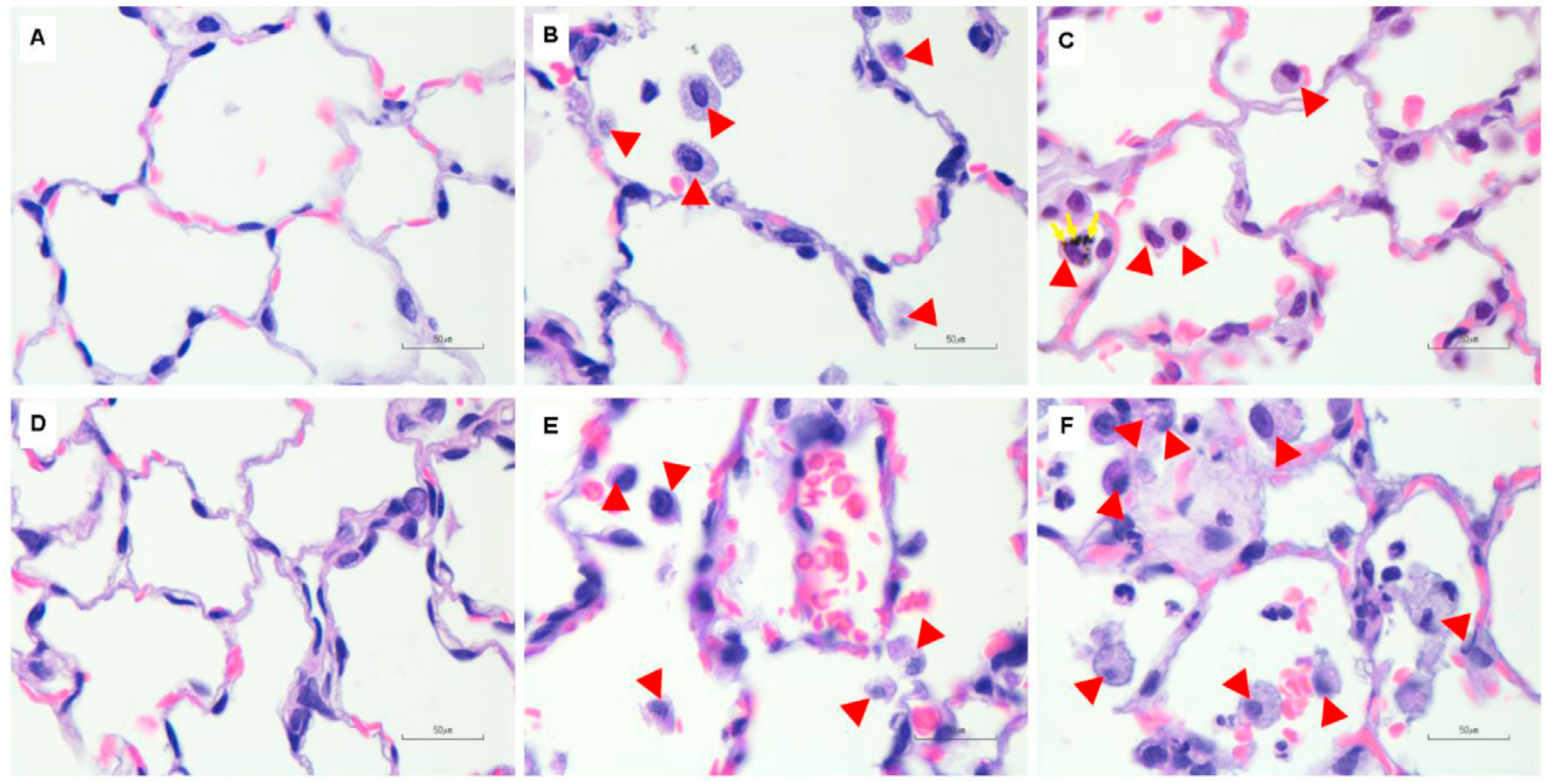
Representative images of alveolar macrophage count in rat lungs using H&E staining (100X, n = 5 per group). (A,D) Alveolar macrophage count of control rats after being exposed to room air for 7 and 14 days. (B,E) Non-filtered CSE groups and (C,F) filtered CSE groups at 7 and 14 days showed small clusters of macrophages in the alveolar space. Red arrows indicate alveolar macrophages in the alveolar space of rat lung tissues and solid line arrows indicate particulate matter uptake by alveolar macrophages.

All histopathological results were shown in Table 1. Cigarette smoke exposure with filter and no-filter altered lung pathological parameters, such as tracheal epithelial cells and peribronchiolar epithelial cell proliferation on day 7 and day 14. Rats in cigarette smoke exposure groups with and without filter showed a significant (p < 0.05) increase in tracheal epithelial cell proliferation on day 14 but not on day 7 (Figure 2). Peribronchiolar epithelial cell proliferation significantly (p < 0.05) increased in the no-filter group on day 7 and 14 when compared to control and filter groups (Figure 3). Lung parenchymal infiltration had an upward trend in both cigarette smoke groups as compared with the control group (Figure 4). In addition, alveolar macrophage counts also significantly (p < 0.05) increased in both cigarette smoke groups on day 7 and 14 when compared with the control group (Figure 5).

### 3.2 Effects of cigarette smoke exposure with filter and no-filter on VDR distribution levels in cytoplasm and nucleus of rat lungs

**Figure 6:**
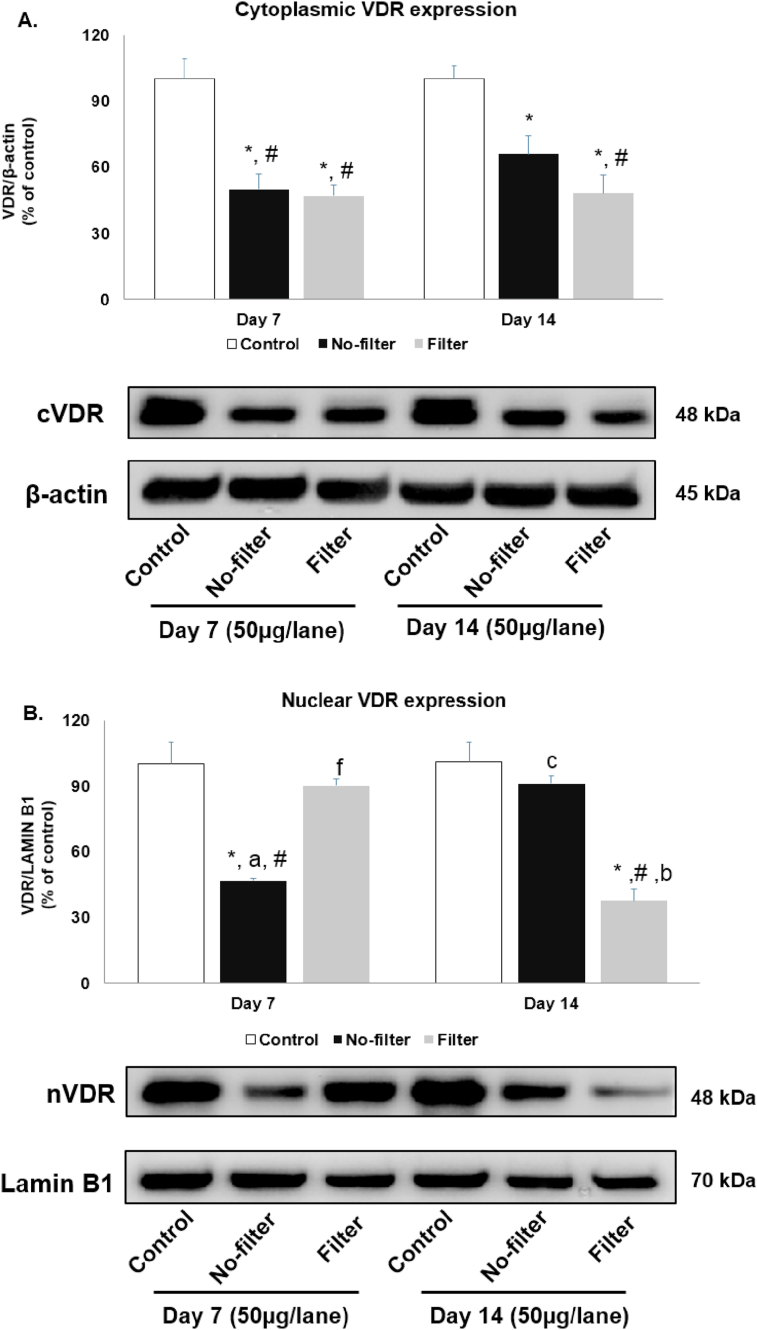
Western blot analyses of VDR distribution from cytoplasmic and nuclear fractions in rat lungs. (A) Cytoplasmic (cVDR) and (B) nuclear VDR (nVDR) protein expression in rat lungs were shown in bar charts and band densities (n = 5 for each group). β-actin and lamin B1 were used as loading control. ∗, p < 0.05 compared with the control group at 7 days. #, p < 0.05 compared with control group at 14 days. a, p < 0.05 comparison between no-filter and filter groups at 7 days. b, p < 0.05 comparison between no-filter and filter groups at 14 days. c, p < 0.05 comparison between no-filter groups at 7 and 14 days. f, p < 0.05 comparison between filter groups at 7 days and 14 days. Data were expressed as mean ± standard error of mean. Full versions of all images are provided in Supplementary figure 1 and 2.

Right lung was used to determine the effects of cigarette smoke exposure on VDR distribution between cytoplasm and nucleus. Cytoplasmic VDR distribution significantly (p < 0.05) decreased in no-filter and filter groups at day 7 and day 14 when compared with both time points of control groups. Cytoplasmic VDR distribution was notably lower in filter group than no-filter group at day 14, while it was similar between the two groups at day 7 (Figure 6A).

Similarly, level of nuclear VDR distribution significantly (p < 0.05) declined after smoke exposure in no-filter group at day 7 when compared to both time points of control groups. Nuclear VDR distribution was also lower in no-filter group at day 7 than in filter group at day 7 and no-filter group at day 14. On the other hand, in filter group, nuclear VDR distribution was unchanged at day 7 but significantly (p < 0.05) lower at day 14 when compared with control groups at day 7 and 14, filter group at day 7 and no-filter group at day 14 (Figure 6B).

### 3.3 Effects of cigarette smoke exposure with filter and no-filter on ERK protein activation in cytoplasm and nucleus of rat lungs

**Figure 7:**
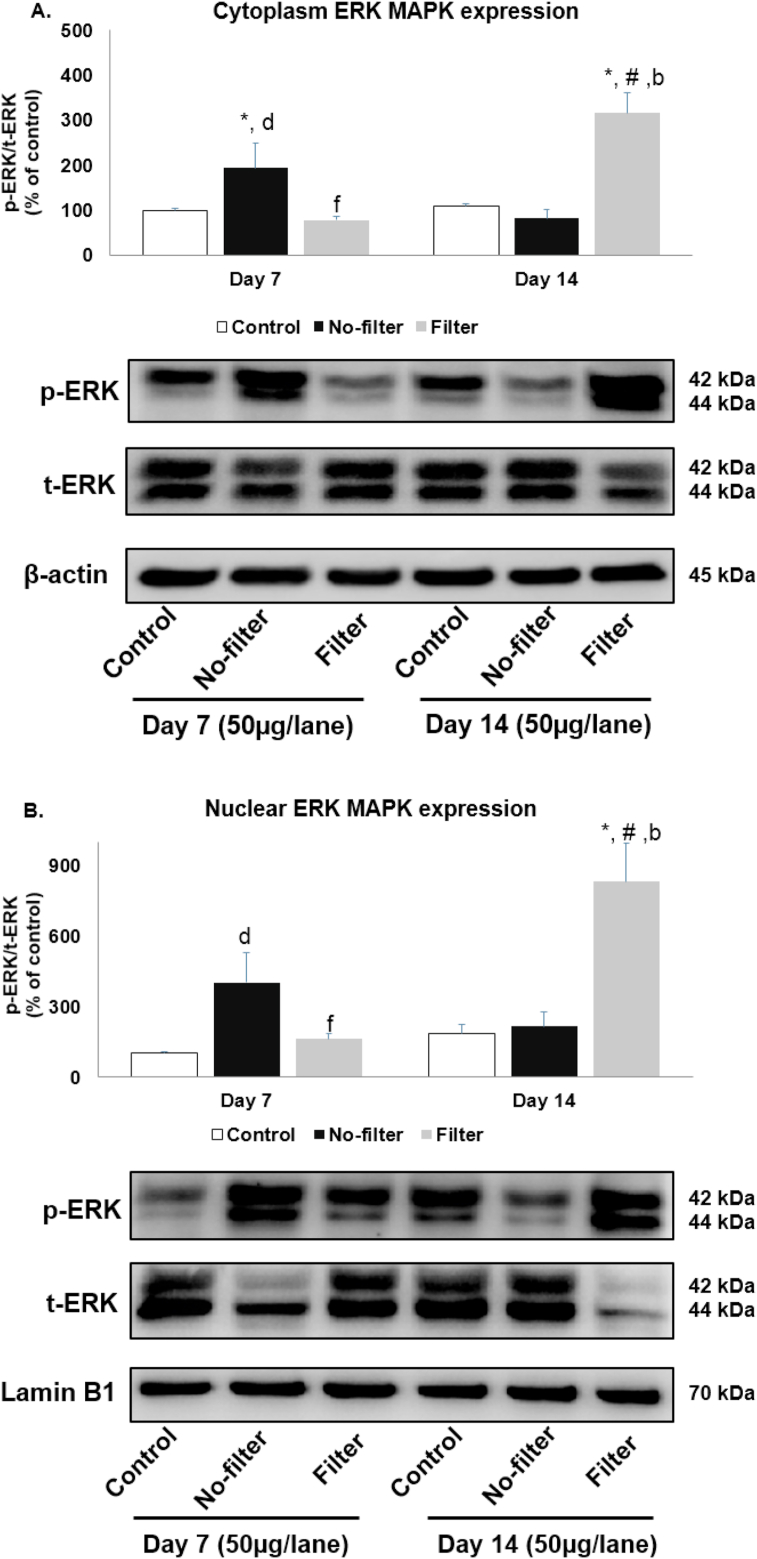
Western blot analyses of ERK distribution from cytoplasmic and nuclear fractions in rat lungs. Extracellular regulated kinase (ERK) protein expression of (A)cytoplasmic and (B) nuclear parts were shown in bar charts and band densities (n = 5 for each group). β-actin and lamin B1 were used as loading control. ∗, p < 0.05 compared with the control group at 7 days. #, p < 0.05 compared with the control group at 14 days. b, p < 0.05 comparison between no-filter and filter groups at 14 days. d, p < 0.05 comparison between no-filter group at 7 days and filter group at 14 days. f, p < 0.05 comparison between filter groups at 7 and 14 days. Data were expressed as mean ± standard error of mean. Full versions of all images are provided in Supplementary figure 3 and 4.

Band densities of phosphor and total ERK protein isoform at 44 kDa were expressed as a ratio of phosphor per total proteins and calculated to the percentage of control by normalization with proteins β-actin and lamin B1. Cytoplasmic ERK activation significantly (p < 0.05) increased in the no-filter group at day 7 as compared with the control group of the same time point and declined at day 14. Cytoplasmic ERK activation in the no-filter group at day 14 was significantly (p < 0.05) lower when compared with the filter group of the same time point. Contrarily, cytoplasmic ERK activation in the filter group was similar to the control group at day 7 but significantly (p < 0.05) upregulated at day 14 when compared with control groups at both time points, filter group at day 7, and no-filter group at day 14 (Figure 7A). The levels of nuclear ERK expression exhibited the same pattern of protein activation in all experiment groups as their cytoplasmic counterparts (Figure 7B).

### 3.4 Effects of cigarette smoke exposure with filter and no-filter on JNK protein activation in cytoplasm and nucleus of rat lungs

**Figure 8:**
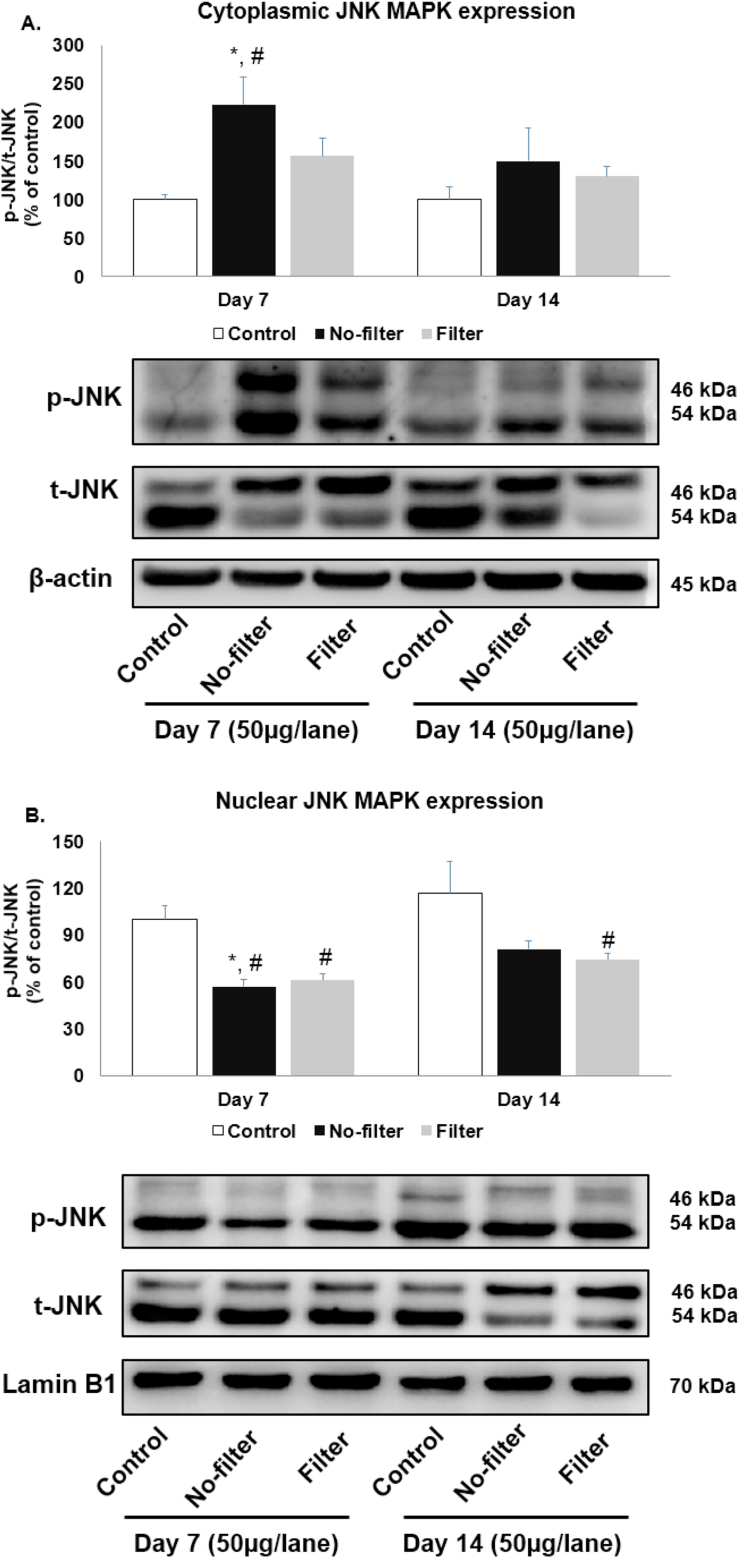
Western blot analyses of JNK distribution from cytoplasmic and nuclear fractions in rat lungs. C-Jun N-terminal kinase (JNK) protein expression of (A) cytoplasmic and (B) nuclear parts were shown in bar charts and band densities (n = 5 for each group). β-actin and lamin B1 were used as loading control. ∗, p < 0.05 compared with the control group at 7 days. #, p < 0.05 compared with the control group at 14 days. Data were expressed as mean ± standard error of mean. Full versions of all images are provided in Supplementary figure 5 and 6.

Band densities of phosphor and total JNK protein isoform at 46 kDa were expressed as a ratio of phosphor per total proteins and calculated to the percentage of control by normalization with proteins β-actin and lamin B1. Cytoplasmic JNK protein activation significantly (p < 0.05) increased in the no-filter group at day 7 compared to both control groups and declined to the levels resembled those of controls at day 14. In filter group, protein levels of JNK were slightly higher at day 7 and 14 compared to their respective control groups, albeit not statistically significant (Figure 8A). Nuclear JNK protein activation decreased in the no-filter group at day 7 and day 14 when compared with both control groups but the differences were only significant at day 7 but not at day 14. For filter groups, JNK protein levels were lower at day 7 and day 14 but the differences only reached statistical significance when compared with the control group at day 14 (Figure 8B).

### 3.5 Effects of cigarette smoke exposure with filter and no-filter on p38 protein activation in cytoplasm and nucleus of rat lungs

**Figure 9:**
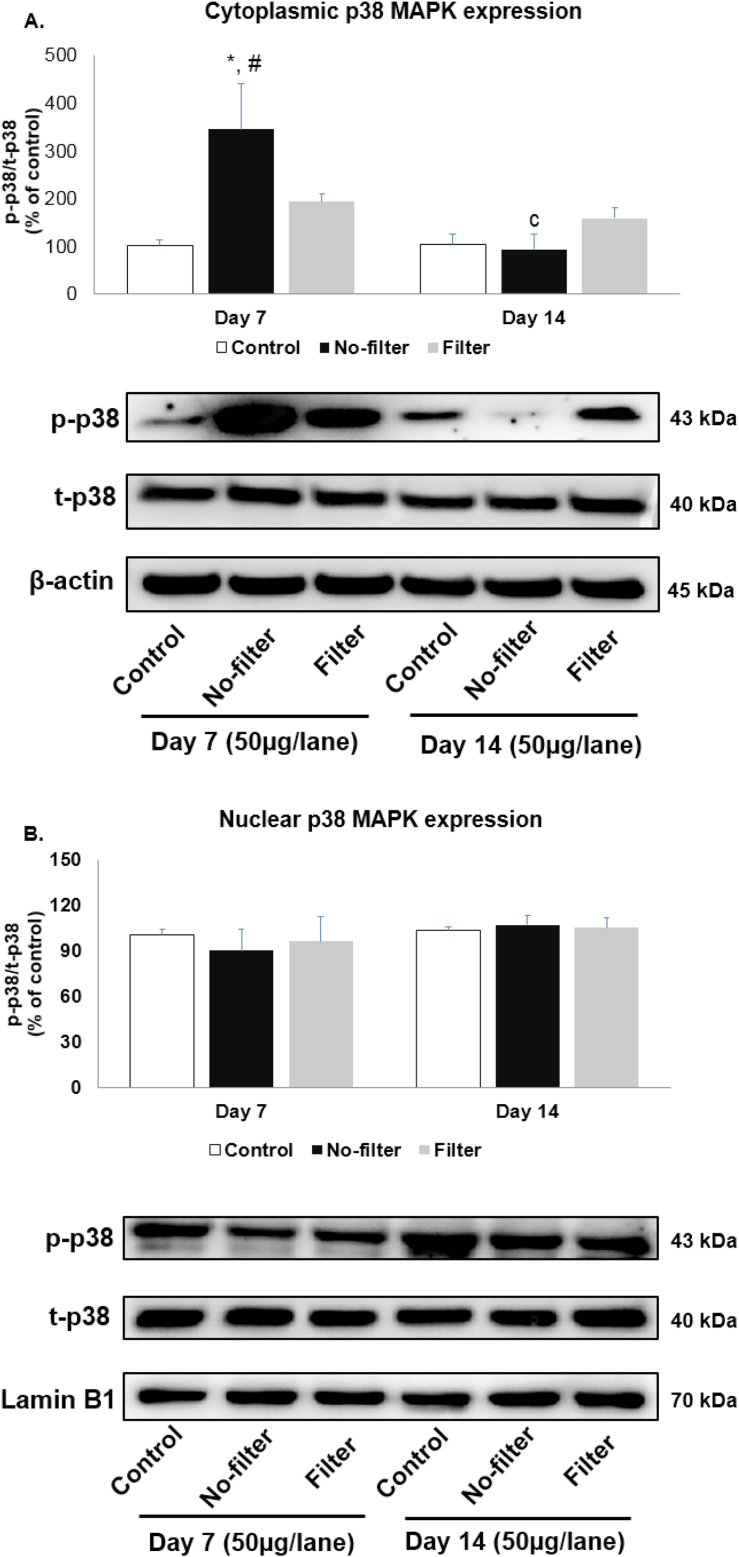
Western blot analyses of p38 distribution from cytoplasmic and nuclear fractions in rat lungs. p38 protein expression of (A) cytoplasmic and (B) nuclear parts were shown in bar charts and band densities (n = 5 for each group). β-actin and lamin B1 were used as loading control. ∗, p < 0.05 compared with the control group at 7 days. #, p < 0.05 compared with the control group at 14 days. c, p < 0.05 comparison between no-filter groups at 7 and 14 days. Data were expressed as mean ± standard error of mean. Full versions of all images are provided in Supplementary figure 7 and 8.

After 7 days of smoke exposure, cytoplasmic p38 activation was significantly (p < 0.05) upregulated in the no-filter group when compared with both control groups but non-significantly increased in the filter group. At day 14, cytoplasmic p38 expression declined in both filter and no-filter groups compared to their respective groups at day 7; however, the differences were only significant in the no-filter group (Figure 9A). On the contrary, nuclear p38 MAPK levels were similar among all experiment groups (Figure 9B).

### 3.6 Effects of cigarette smoke exposure on VDR localization in rat lungs

**Figure 10:**
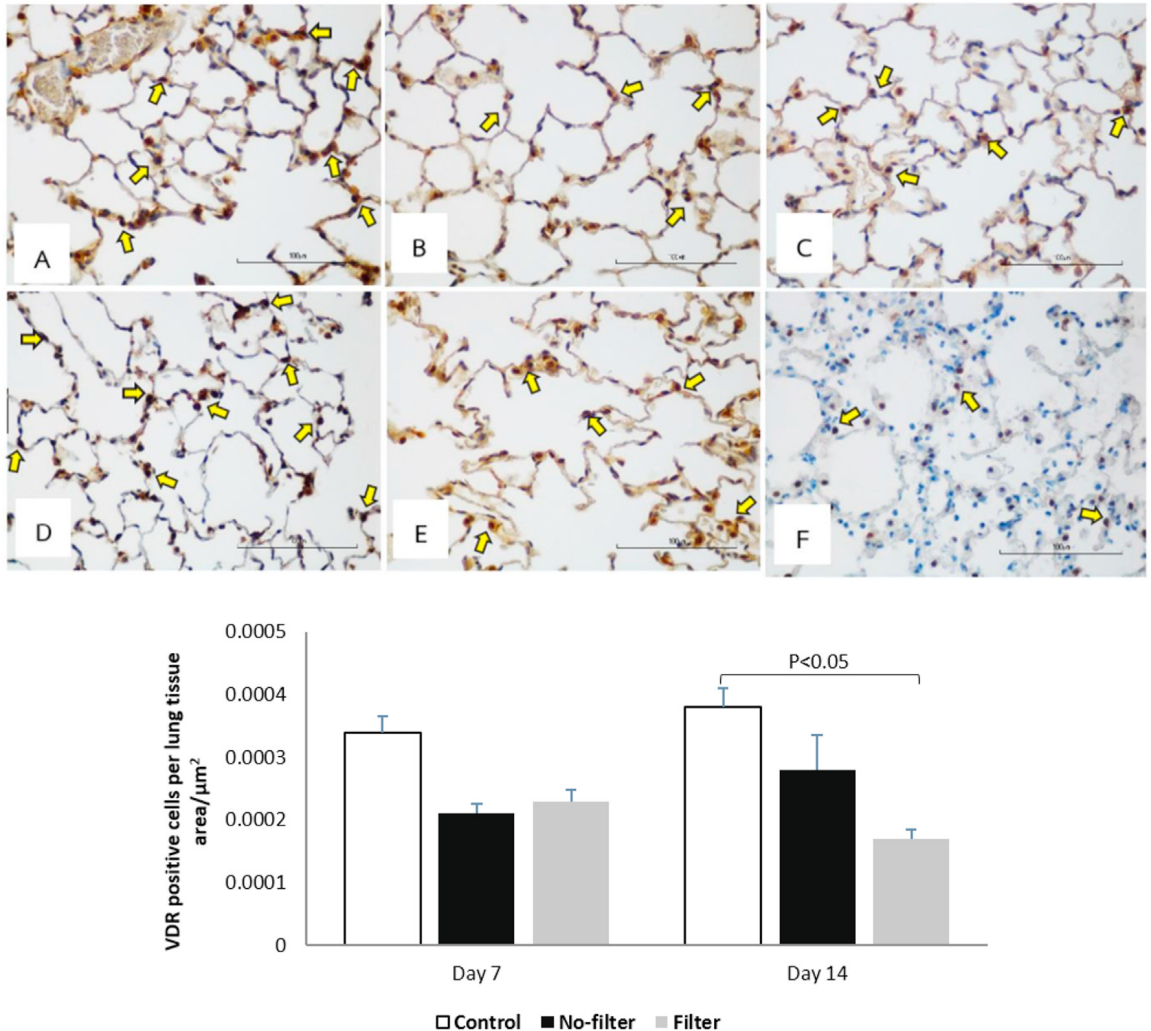
Immunohistochemistry of VDR distribution in rat lungs (40X, n = 5 per group). (A,D) Control groups at 7 and 14 days, (B,E) No-filter groups at 7 and 14 days, and (C,F) filter groups at 7 and 14 days, respectively. VDR expression were shown in histological images and bar charts. Arrows indicated VDR positive pneumocytes type II cells with dark brown stained nuclei. p < 0.05 when compared the filter group at 14 days with the control group of the same time point. Data were expressed as mean ± standard error of mean.

To investigate the localization of VDR in rat lungs, the immunohistochemistry analysis was used in this study. Generally, VDR expression could be seen on pneumocytes or alveolar type II (AT II) cells in the lung [18]. The immunohistochemistry showed that VDR was highly expressed in control groups at both time points. After 7 days of cigarette smoke exposure, VDR had a trend toward decreased expression in both no-filter and filter groups. VDR expression after 14 days of CSE increased in the non-filter group but declined further in the filter group. As a result, VDR expression was significant lower in the filter group at day 14 when compared to the control group of the same time point (Figure 10).

## 4 Discussion

In this study, we attempted to explicate the effects of cigarette smoke exposure with and without filter on VDR and MAPKs cascade distribution at the subcellular levels and its relation to lung pathogenesis by using a rat model that imitated human smoking behavior. We found that smoke exposure in acute (7 days) and subacute (14 days) phases could induce changes in all lung parameters which included tracheal epithelial cell changes, peribronchiolar epithelial cell proliferation, lung parenchymal infiltration and increased alveolar macrophages. These parameters were used as histopathological markers of lung tissue injury leading up to the development of emphysema/COPD [19, 20].

Vitamin D receptor (VDR) is assumed to be an essential part of lung pathogenesis. The distribution of cytoplasmic and nuclear VDR is relevant to distinct biological activities, such as transcriptional regulation, immune regulatory process, anti-microbial activity and anti-cancer effects [8]. Previous studies reported that decreased VDR expression was associated with lower survival and higher disease progression in several neoplastic conditions [21, 22, 23, 24]. However, the change in cytoplasmic and nuclear VDR distribution at subcellular levels in the context of early cigarette smoke exposure *in vivo* has rarely been explored. To the best of our knowledge, this is also the first study to evaluate the effects of commercial cigarette filter on subcellular responses to cigarette smoke exposure. Regardless of the filter, we found that cytoplasmic VDR expression decreased both at day 7 and day 14 of CSE. On the contrary, nuclear VDR expression decreased at day 7 and returned to normal at day 14 for the no-filter group, while its expression changed in an opposite direction for the filter group. Immunohistochemistry study of VDR expression showed a decline in its expression after 7 and 14 days of CSE but more significantly at day 14 for the non-filter group. We hypothesized that the improved nuclear VDR expression at day 14 in the non-filter group was due to higher ventilation and lower toxic substance accumulation in the absence of filter which allowed protein recovery from noxious stress. On the contrary, both nuclear and cytoplasmic VDR expression in the filter group significantly decreased on day 14 possibly because of toxicant accumulation from low filter ventilation that may further disrupt protein viability [11, 25]. Previous *in vitro* studies demonstrated that CSE interrupted vitamin D induced VDR translocation from nucleus to cytoplasm leading to the reduction in cytoplasmic VDR and decreased VDR mRNA levels in A549 cells. The decline in VDR expression was also associated with increased inflammatory cytokine expression [11, 12]. Similar to their findings, we observed a decrease in VDR expression after CSE especially in the filter group. A recent review article suggested that the reduction of cigarette filter ventilation from cigarette design change could induce lung adenocarcinoma. The incomplete combustion inside the filter generated a large puff volume resulting in increasing particle size and exposing more toxicants to peripheral lung area. Thus, cigarette filter ventilation could be the important factor that increases more toxic substance exposure to alveolar cells and prevents ligand VDR binding to its receptor in both nucleus and cytoplasm [25, 26, 27].

MAPKs are essential messengers of cell-cell communication through the fluctuation of protein interaction in facilitating the spatial and temporal change in cellular organism because these signaling cascades play a role in regulating various cellular processes and reflect insight in their physiological responses [28]. The activation of ERK1/2 has been implicated in cellular proliferation and survival which can be activated by a variety of external stimuli, such growth factors, transforming agents, G protein-coupled receptors and carcinogens based on cell types. In the resting stage, ERK cascades are mainly located in the cytoplasm, and then translocated to the nucleus upon activation [29, 30]. In this study, both cytoplasmic and nuclear ERK expression increased at day 7 and reverted back to normal at day 14 in the no-filter group. On the contrary, both cytoplasmic and nuclear ERK expression in filter group were normal at day 7 but markedly higher at day 14. Previous studies demonstrated that acute cigarette smoke exposure rapidly activated ERK1/2 with subsequent TNF-α release by macrophages, and had proliferative effects on lung epithelial cells via ERK1/2 pathway [31, 32]. Moreover, ERK could activate early growth response gene-1 (EGR-1), which provided a positive feedback loop responsible for the second peak of ERK1/2 phosphorylation after prolong exposure to cigarette smoke extract *in vitro* [33]. Our findings were consistent with others studies in that ERK expression increased after CSE. Previous *in vivo* studies demonstrated that ERK activation caused lung tissue injury through the activation of apoptosis signaling and through the release of inflammatory cytokines that led to inflammatory cell infiltration, alveolar wall destruction, airspace enlargement and bronchiolar epithelial hyperplasia [16, 34, 35]. Our results confirmed that nuclear ERK activation might be the inducer of macrophage infiltration which could be manifested in an acute phase of CSE and played a role in gene regulation of mitogenic reaction in lung histopathology.

JNK cascade regulated a variety of cellular process, such as apoptosis, inhibition of cell growth, cytokine secretion and inflammation [36, 37]. We found that both exposure types had similar pattern of cytoplasmic and nuclear JNK activation. The no-filter group showed significantly increased cytoplasmic JNK and significantly decreased nuclear JNK at day 7 but both changes became less prominent at day 14. Whereas, the filter group showed non-significant trend toward higher cytoplasmic JNK expression at day 7 and 14 and a significant decrease in nuclear JNK expression at day 7 and 14. A previous publication suggested that cytoplasmic JNK activation exerted an anti-proliferative effect through the inhibiting of JNK gene regulated cellular proliferation in the cytoplasm via the JNK interacting protein-1 (JIP-1) [38]. A recent study demonstrated that cigarette smoke exposure increased protein phosphatase 2A (PP2A) activity, which in turn inactivated JNK. This was believed to be a defensive mechanism against CSE induced inflammatory responses. It is important to note, however, that PP2A did not alter ERK or p38 activation. Thus, sustained inflammation following CSE could still happen through other pathways [39, 40]. Increased PP2A activity after cigarette exposure might explain the reduction in nuclear translocation on JNK seen in our study.

The activation of p38 stimulates the production of cytokines in immune cells which are mediated by both specific kinases MKK3 and MKK6 [41,42]. There is still uncertainty regarding the role of p38 subcellular localization after early cigarette smoke exposure. In this study, cytoplasmic p38 expression in the no-filter group was significantly upregulated at day 7 and reverted back to normal level at day 14. The similar pattern of cytoplasmic p38 expression changes was seen in the filter group but differences were not significant. Nuclear p38 expression, however, was not affected by CSE regardless of the filter. In accordance with our study, CSE has been shown in both *in vitro* and *in vivo* studies to activate p38 MAPK which led to lung injury through the increase in neutrophil infiltration, proteinase expression, apoptosis, and oxidative DNA damage [16, 43]. Furthermore, MAPKAP kinase-2 was a nuclear localize signal (NLS) that cooperated with p38 conformational change for the activation in the nucleus and then relocated to the cytoplasm to phosphorylate their substrates during stress response [44]. Currently available evidence suggests that cytoplasmic p38 might have the pivotal role in the development of lung tissue injury following CSE. However, from this study, we did not have the information regarding which cells were affected by p38 expression changes. Further studies with immunohistochemical studies are warranted.

In conclusion, acute and subacute cigarette smoke exposure with no-filter and filter caused similar pathological changes in the lung. In the subcellular level, cigarette smoke exposure decreased VDR expression and increased ERK expression but the effects were delayed and to a greater extent in the filter group. Cigarette smoke exposure regardless of filter decreased nuclear translocation of JNK. The overall effect of cigarette filter did not appear to be protective against lung injury and may be even more detrimental when it came to VDR expression.

## Declarations

### Author contribution statement

F. Okrit: Conceived and designed the experiments; Performed the experiments; Analyzed and interpreted the data; Wrote the paper.

P. Chatranuwatana: Performed the experiments; Analyzed and interpreted the data.

D. Werawatganon: Analyzed and interpreted the data; Contributed reagents, materials, analysis tools or data.

M. Chayanupatkul: Analyzed and interpreted the data; Wrote the paper.

S. Sanguanrungsirikul: Conceived and designed the experiments; Analyzed and interpreted the data.

### Funding statement

This work was supported by the 90th Anniversary of Chulalongkorn University, Ratchadapisek Somphot Fund and Ratchadapisek Somphot Fund, Faculty of Medicine, 10.13039/501100002873Chulalongkorn University, grant number 2560-023.

### Data availability statement

Data included in article/supplementary material/referenced in article.

### Competing interest statement

The authors declare no conflict of interest.

### Additional information

No additional information is available for this paper.

## Appendix A Supplementary data

Supplementary data_R6
