# Supplementary material for: Changes of vitamin D receptors (VDR) and MAPK activation in cytoplasmic and nuclear fractions following exposure to cigarette smoke with or without filter in rats
Source: Heliyon. 2021 Jan 30;7(1):e05927. doi: 10.1016/j.heliyon.2021.e05927 (PMC7851787; doi:10.1016/j.heliyon.2021.e05927)
Supplement: Supplementary file 1 — Supplementary data_R6 [file mmc1.docx]

**Supplementary data to Okrit et al. “Changes of vitamin D receptors (VDR) and MAPK activation in cytoplasmic and nuclear fractions following exposure to cigarette smoke with or without filter in rats”**

**Supplementary Table 1 Criteria for pathological score assessment (15)**

| Pathological  features  Score | 0: No change | 1: Mild | 2: Moderate | 3: Severe | 4: Very severe |
| --- | --- | --- | --- | --- | --- |
| Tracheal epithelial cell changes | No change | Focal squamous cell metaplasia (S2 met) | Squamous cell metaplasia + hyperplasia | Squamous cell metaplasia +hyperplasia  +acute inflame | Squamous cell metaplasia with hyperplasia + diffuse inflammatory cells |
| Peribronchiolar epithelial cell proliferation | Normal two-layers epithelium | Squamous dysplasia | Mild dysplasia | Moderate dysplasia | Severe dysplasia |
| Lung parenchymal infiltration | No injury | Injury to 25% of the field | Injury to 50% of the field | Injury to 75% of the field | Diffuse injury |
| Alveolar macrophage count | 0 cell | 1-4 cells | 5-9 cells | ≥10 cells | Abundant cells |

**Day 14 (50 µg/lane)**

**Day 7 (50 µg/lane)**

No-filter

Filter

Control

Filter

No-filter

Control


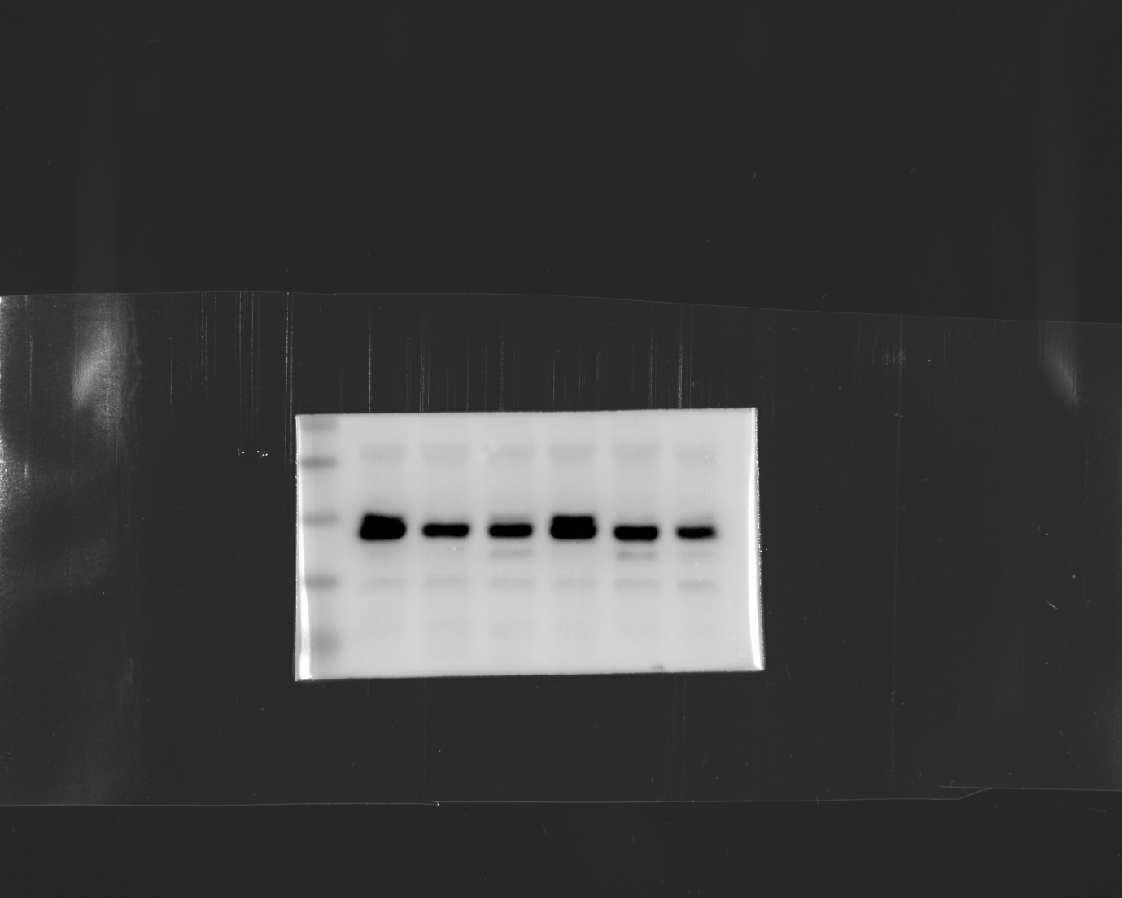


48 kDa

cytoplasmic VDR


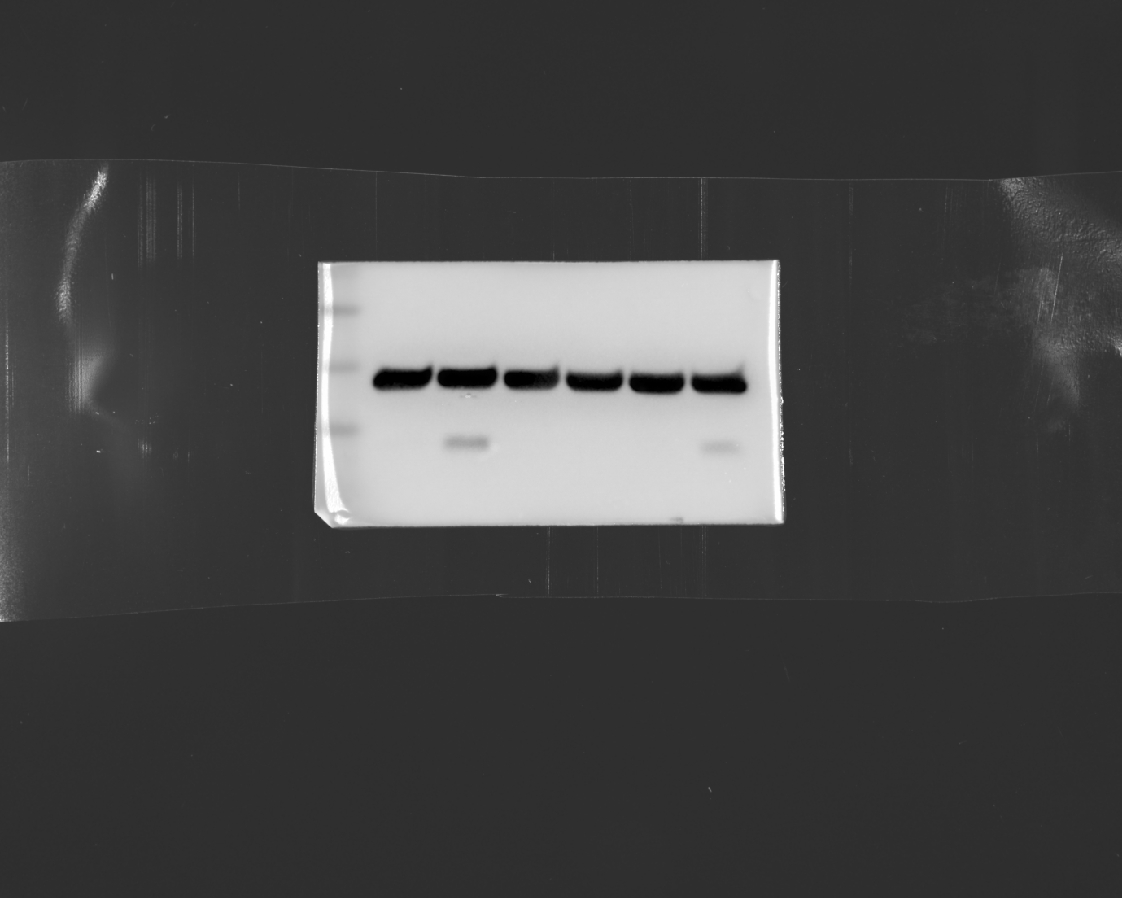


45 kDa

β-actin

**Supplementary Fig. 1** Full western blotting images for cytoplasmic VDR and β-actin band densities. Six middle panels indicate each experimental group at different time points (the first three panels represent control, no-filter and filter groups of day 7, respectively and the last three panels represent control, no-filter and filter groups of day 14, respectively).

**Day 14 (50 µg/lane)**

**Day 7 (50 µg/lane)**

Control

No-filter

Filter

Control

No-filter

Filter


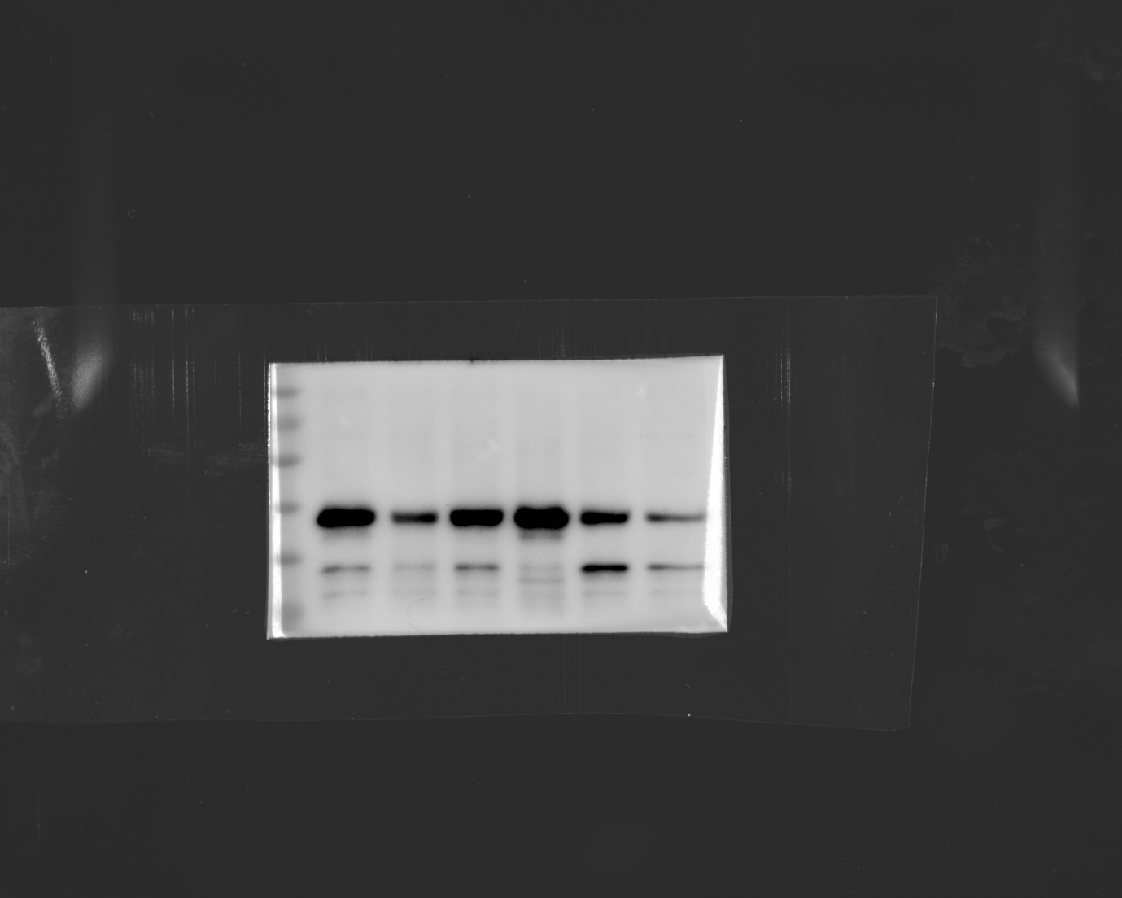


48 kDa

nuclear VDR


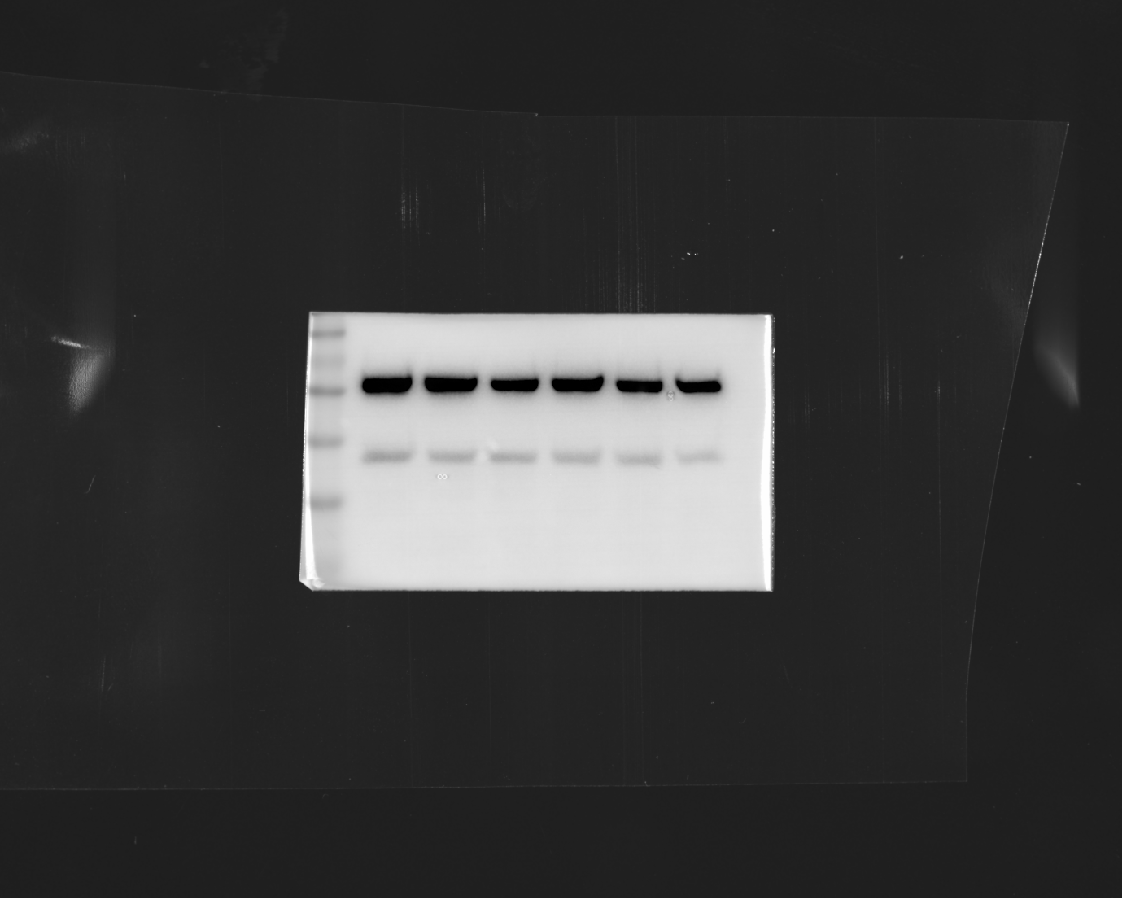


70 kDa

Lamin B1

**Supplementary Fig. 2** Full western blotting images for nuclear VDR and lamin B1 band densities. Six middle panels indicate each experimental group at different time points (the first three panels represent control, no-filter and filter groups of day 7, respectively and the last three panels represent control, no-filter and filter groups of day 14, respectively).

**Day 14 (50 µg/lane)**

Filter

No-filter

Control

Filter

No-filter

Control

**Day 7 (50 µg/lane)**

**
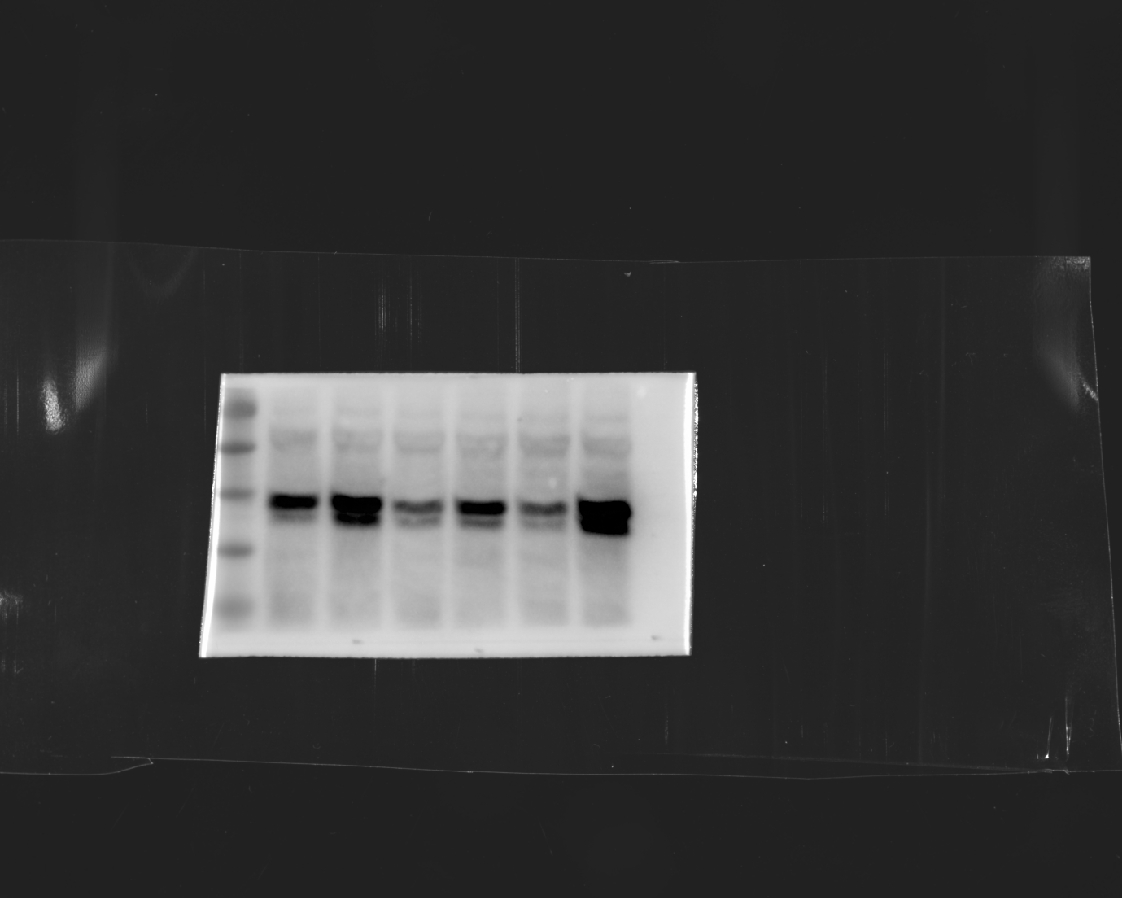
**

42, 44 kDa

Cytoplasmic phospho-ERK

**
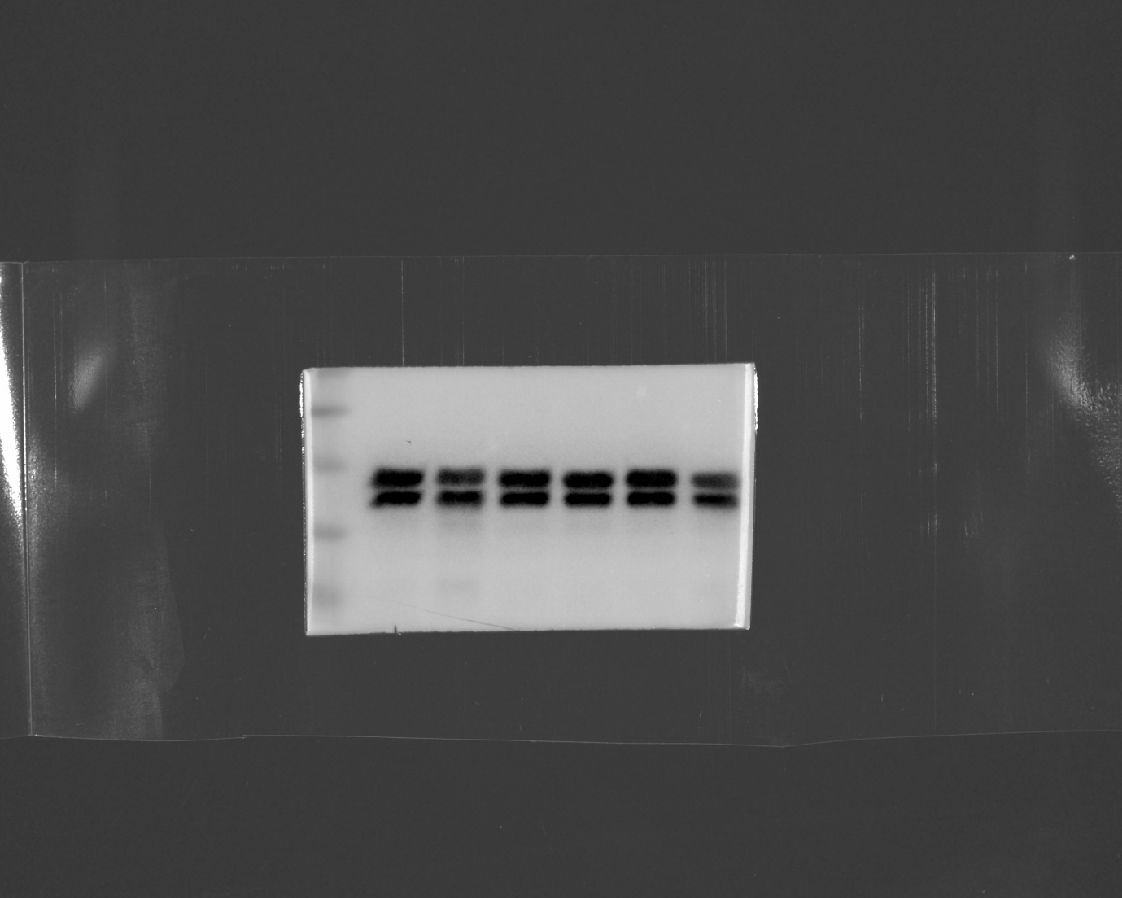
**

42, 44 kDa

Cytoplasmic total-ERK

**
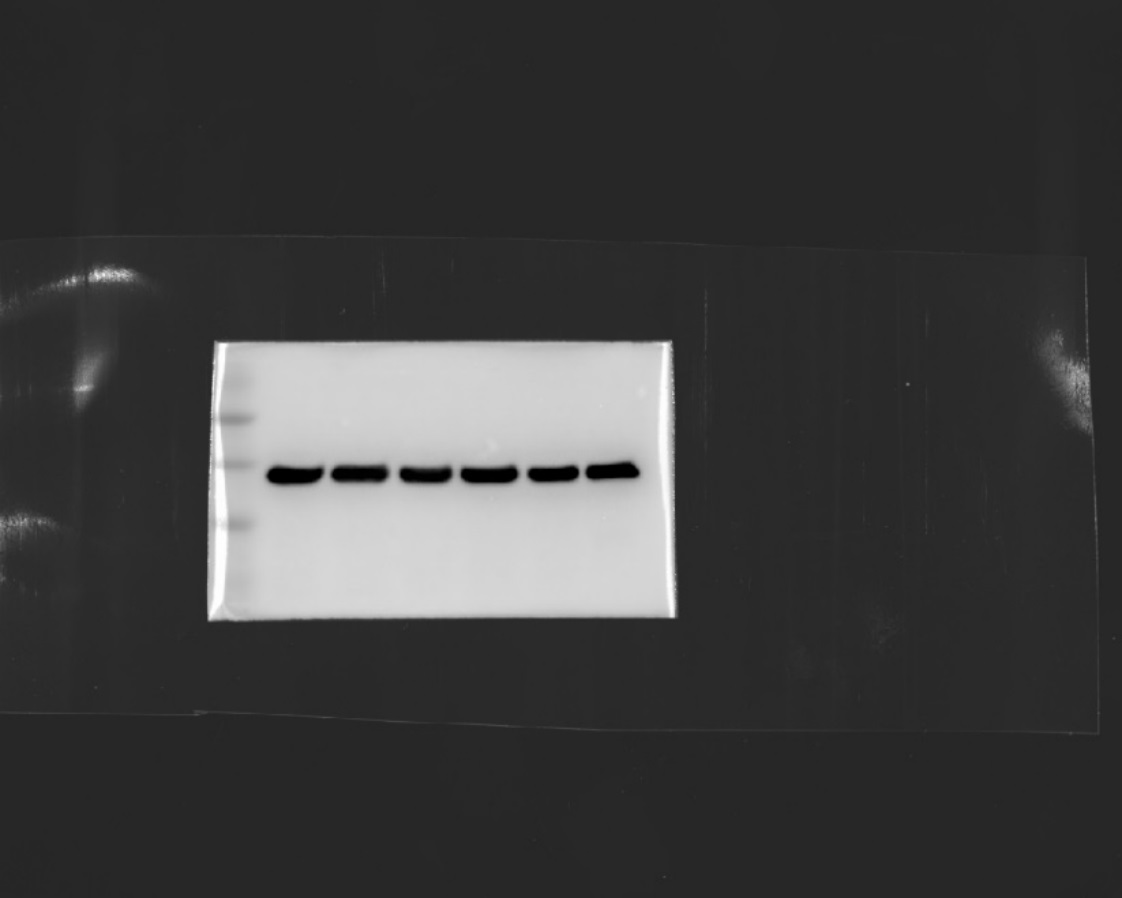
**

45 kDa

β-actin

**Supplementary Fig. 3** Full western blotting images for cytoplasmic phosphor-ERK, total-ERK and β-actin band densities. Six middle panels indicate each experimental group at different time points (the first three panels represent control, no-filter and filter groups of day 7, respectively and the last three panels represent control, no-filter and filter groups of day 14, respectively).

**Day 14 (50 µg/lane)**

**Day 7 (50 µg/lane)**

Control

No-filter

Filter

Control

No-filter

Filter


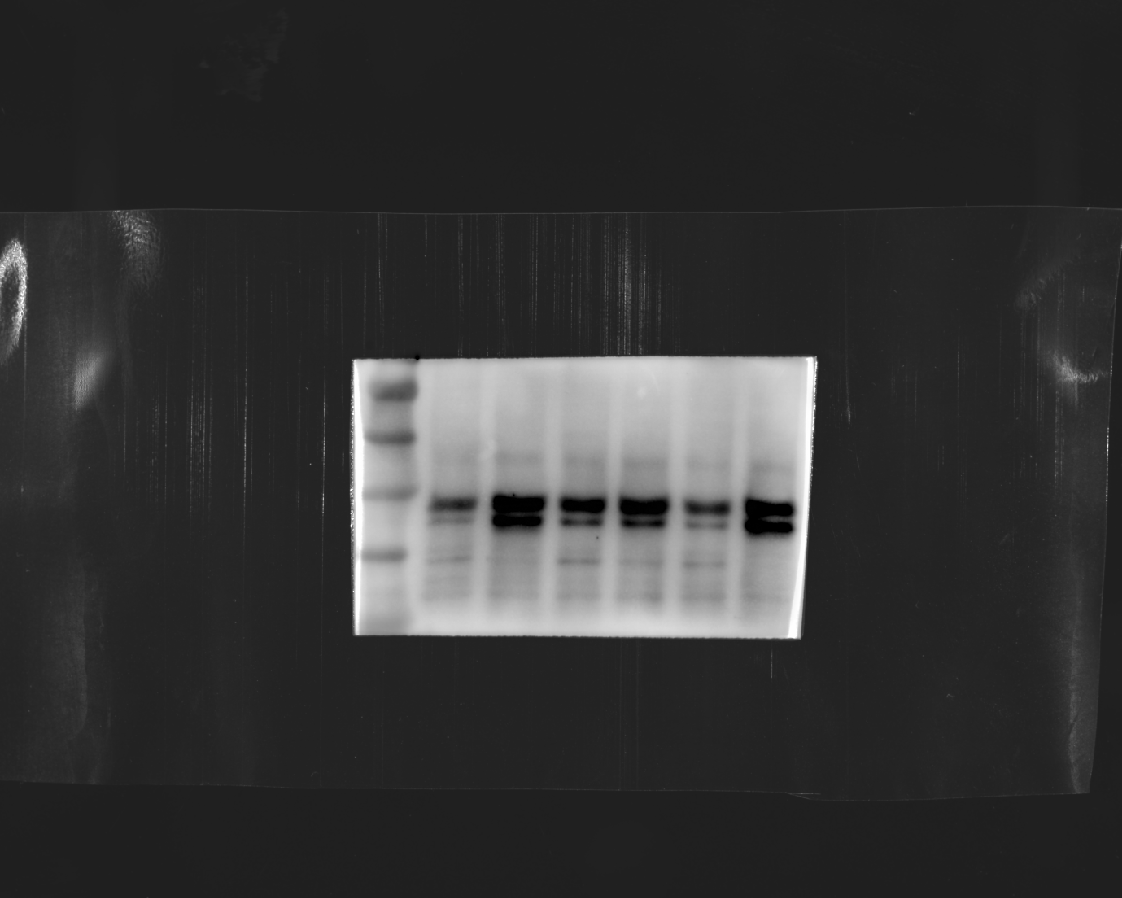


42, 44 kDa

Nuclear phospho-ERK


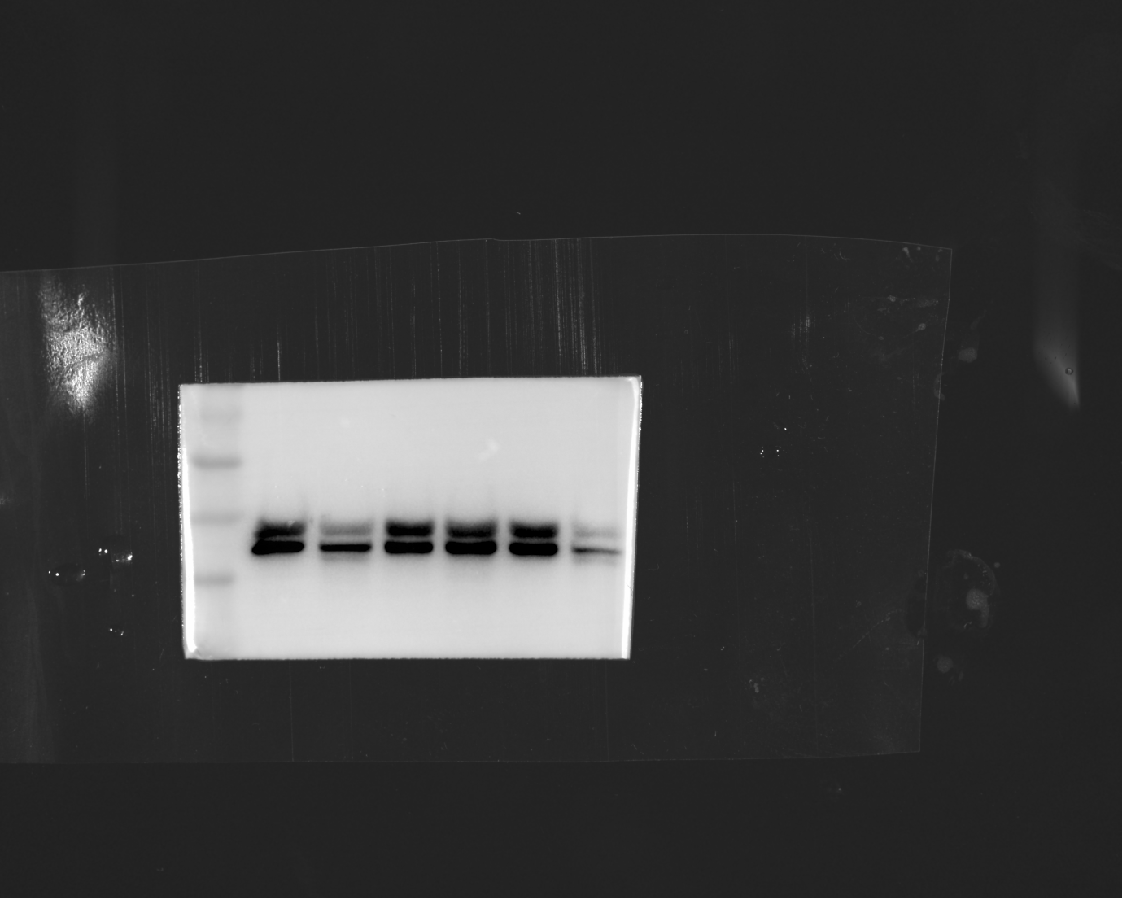


Nuclear total-ERK

42, 44 kDa


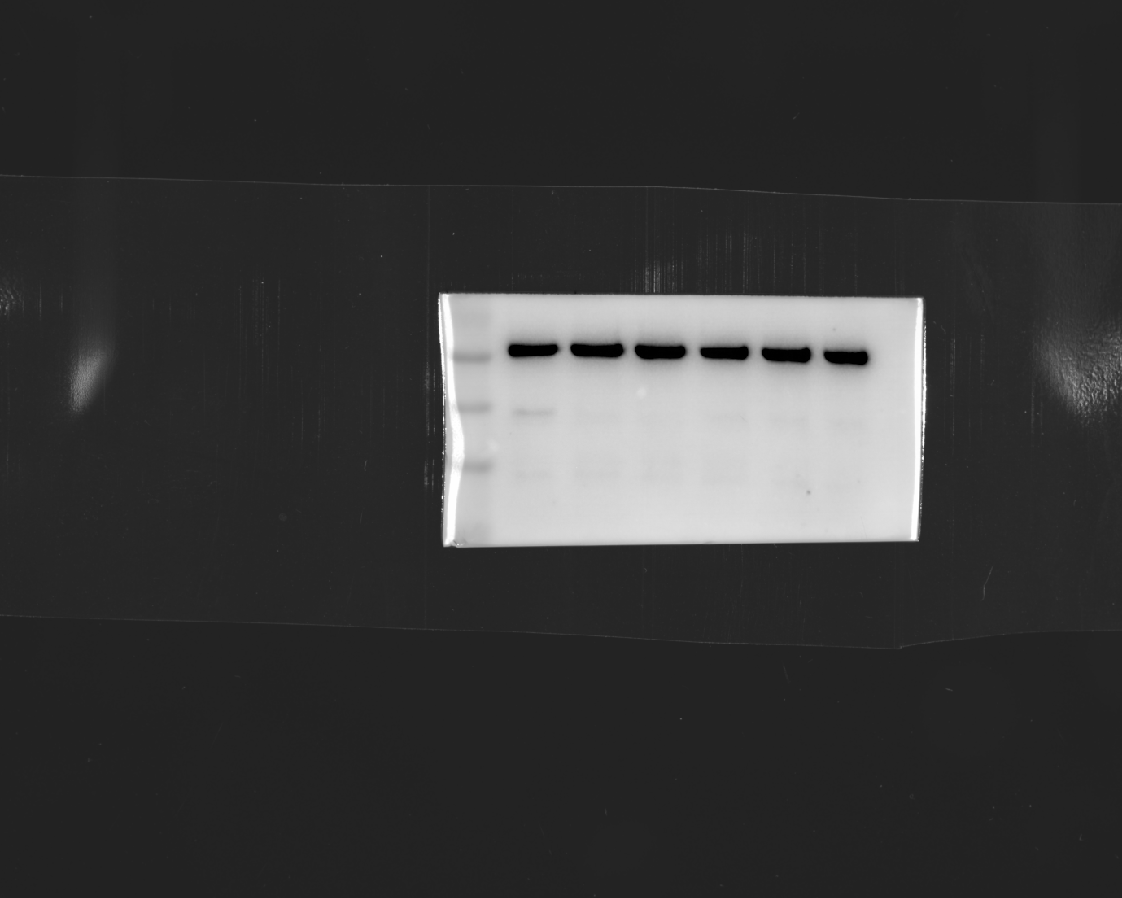


70 kDa

Lamin B1

**Supplementary Fig. 4** Full western blotting images for nuclear phosphor-ERK, total-ERK and lamin B1 band densities. Six middle panels indicate each experimental group at different time points (the first three panels represent control, no-filter and filter groups of day 7, respectively and the last three panels represent control, no-filter and filter groups of day 14, respectively).

**Day 14 (50 µg/lane)**

Filter

No-filter

Control

Filter

No-filter

Control

**Day 7 (50 µg/lane)**

**
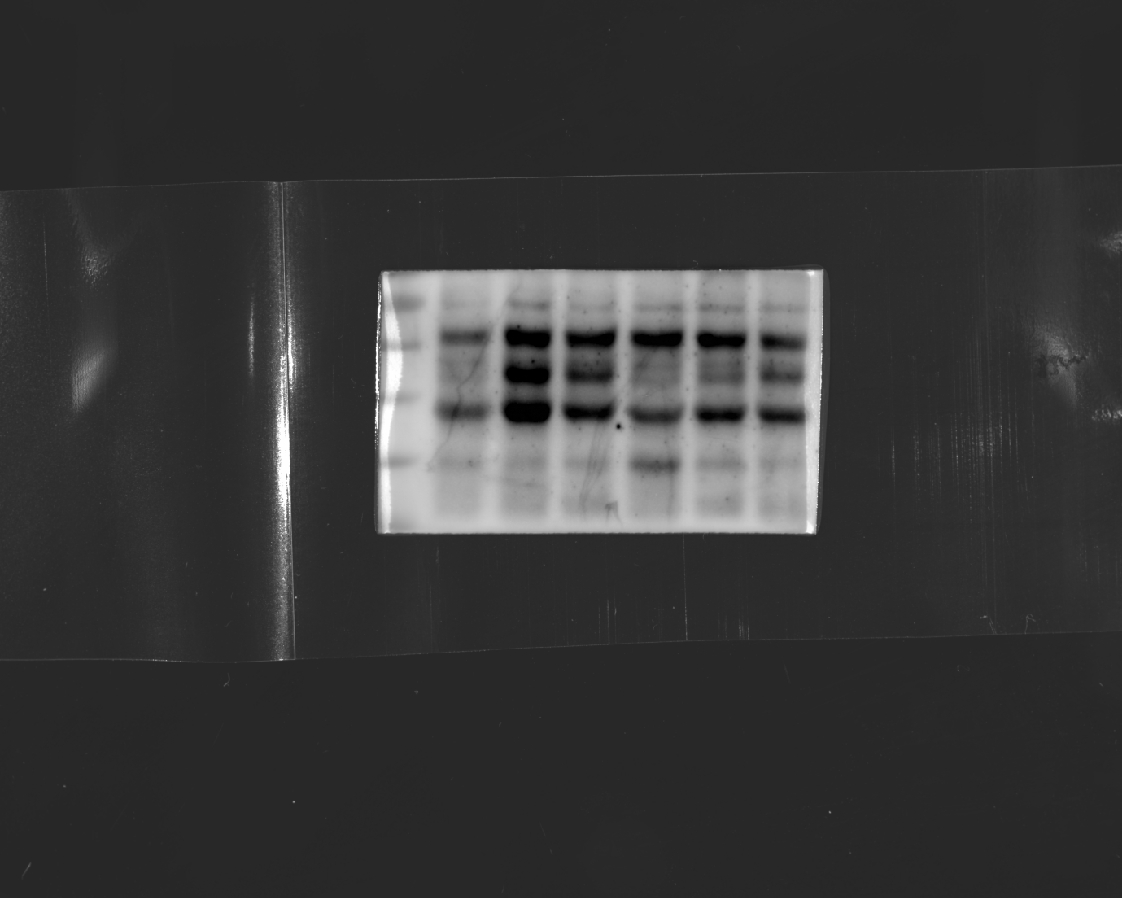
**

46, 54 kDa

Cytoplasmic phospho-JNK


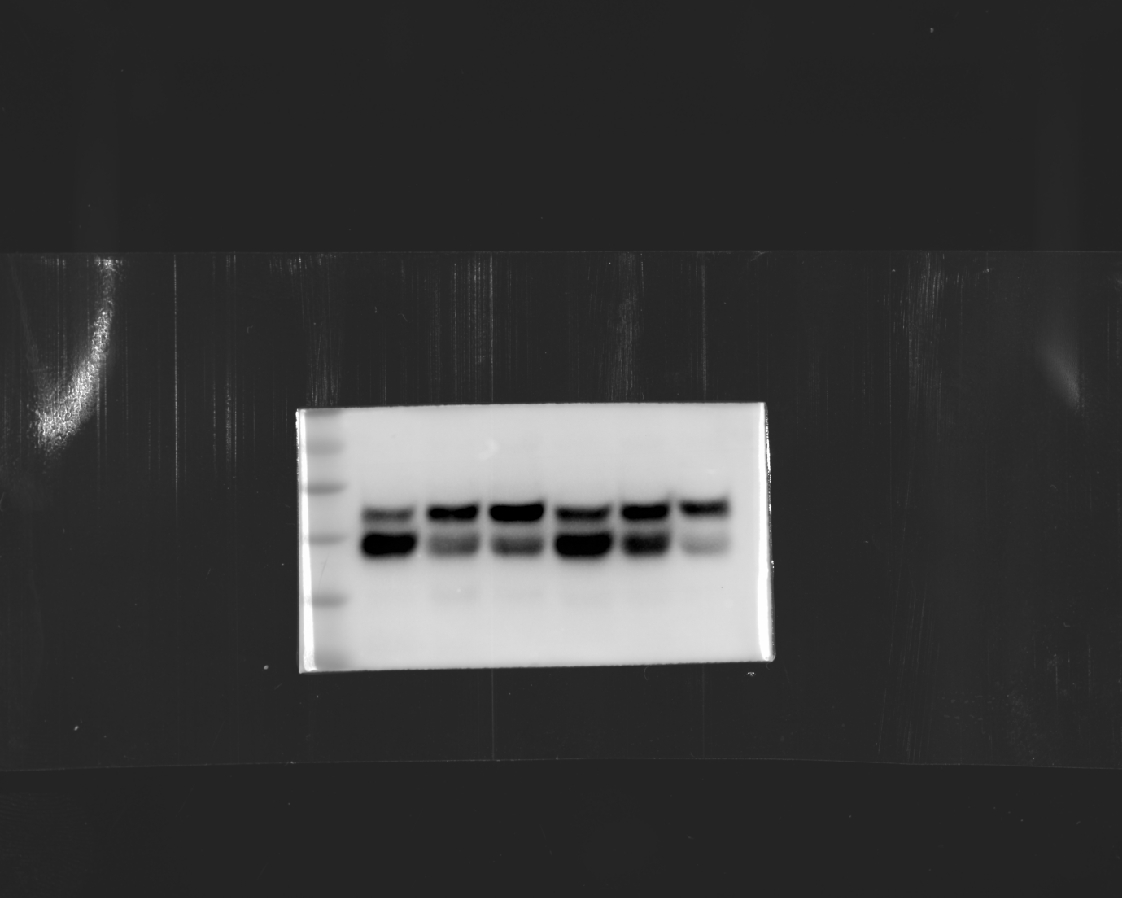


46, 54 kDa

Cytoplasmic total-JNK


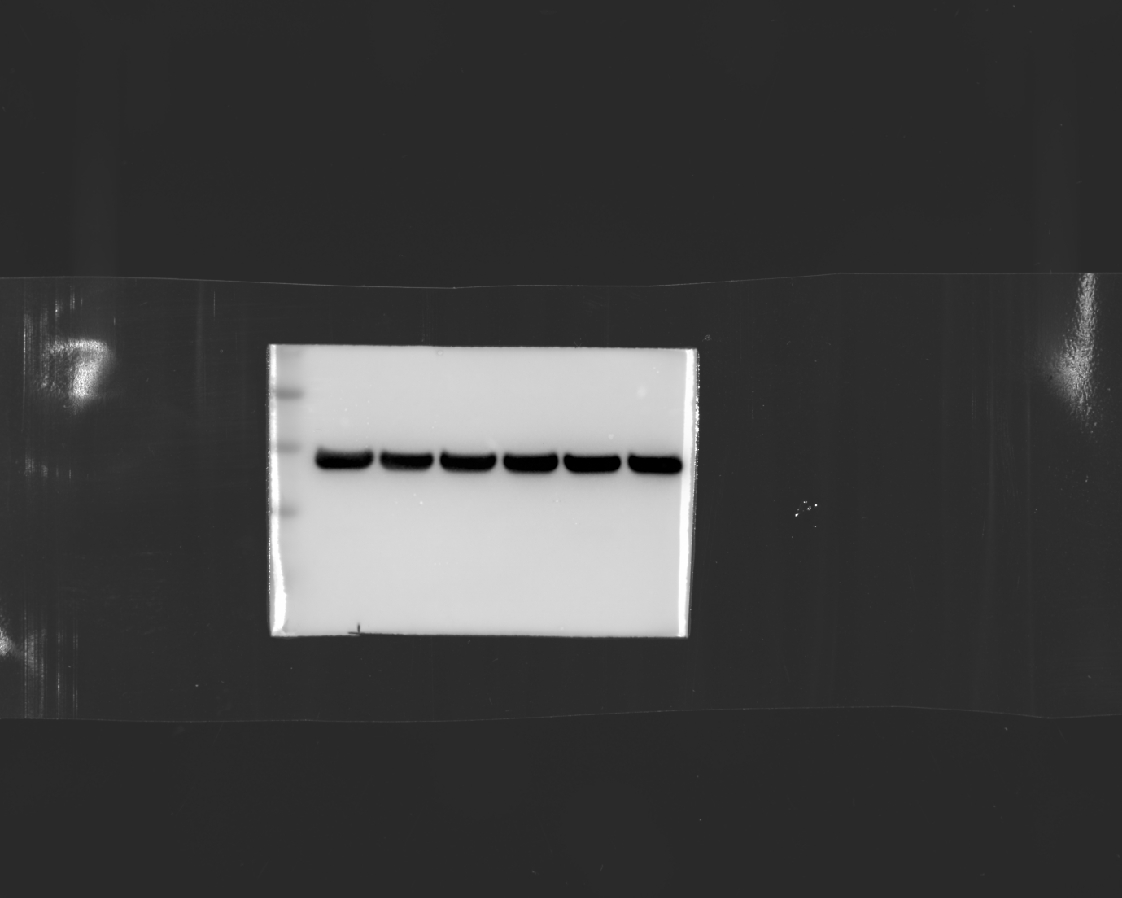


45 kDa

β-actin

**Supplementary Fig. 5** Full western blotting images for cytoplasmic phosphor-JNK, total-JNK and β-actin band densities. Six middle panels indicate each experimental group at different time points (the first three panels represent control, no-filter and filter groups of day 7, respectively and the last three panels represent control, no-filter and filter groups of day 14, respectively). The band panels of cytoplasmic p-JNK above the target molecular weight indicate non-specific band with anti-p-JNK.

**Day 14 (50 µg/lane)**

No-filter

Control

Filter

No-filter

Control

**Day 7 (50 µg/lane)**

Filter


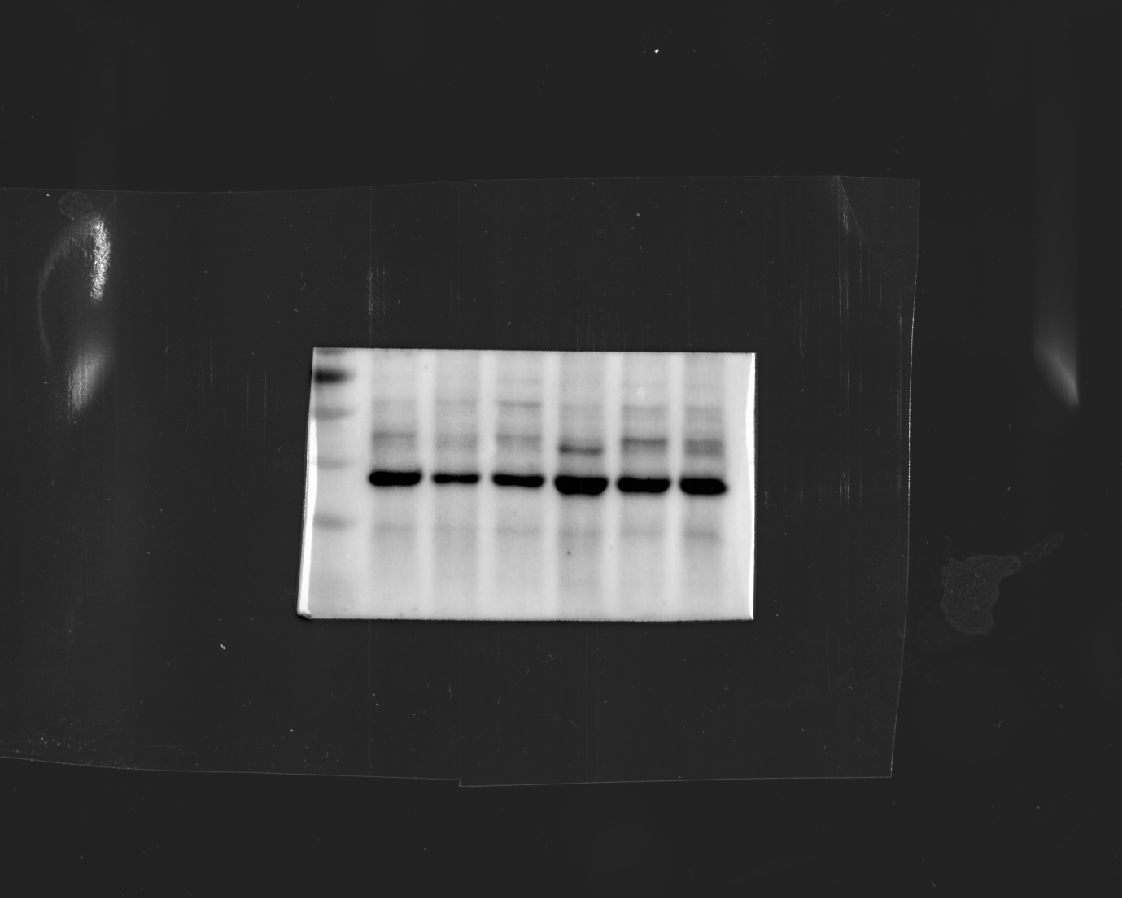


46, 54 kDa

Nuclear phospho-JNK


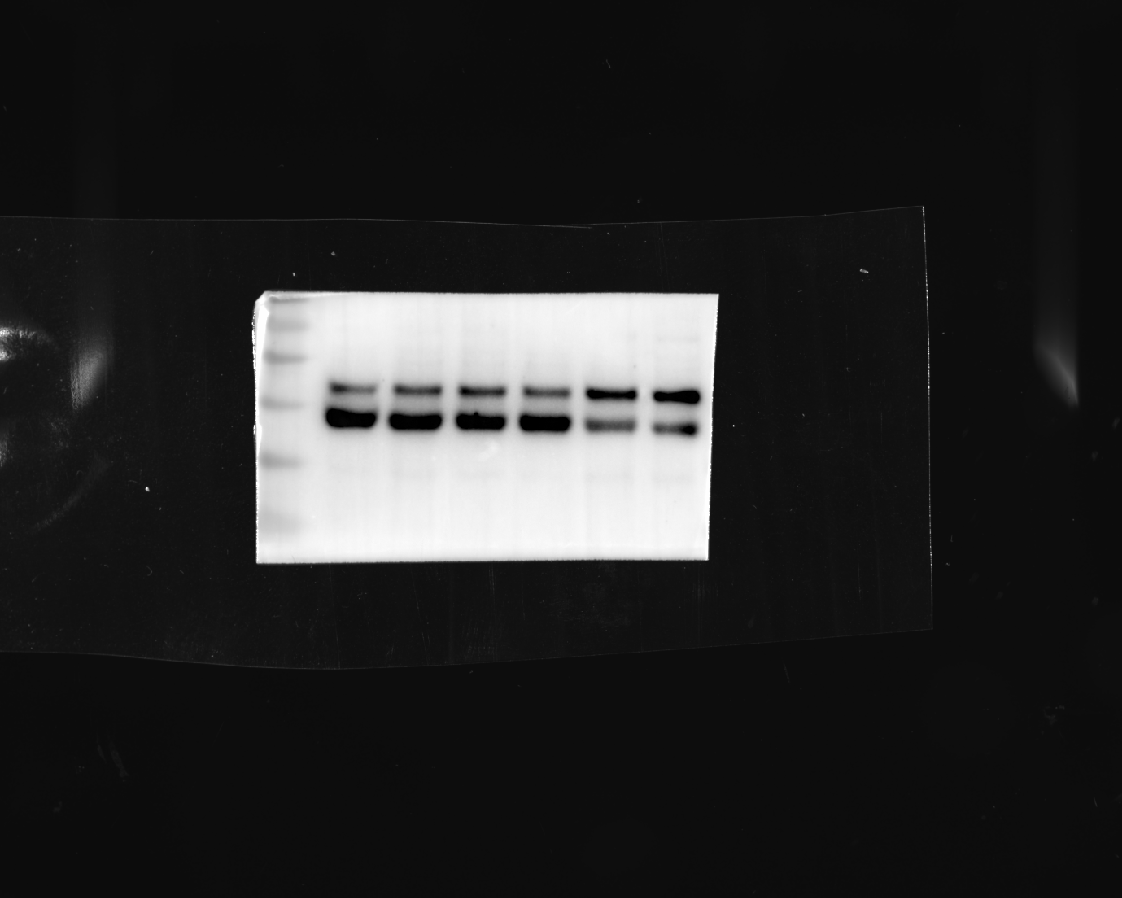


46, 54 kDa

Nuclear total-JNK


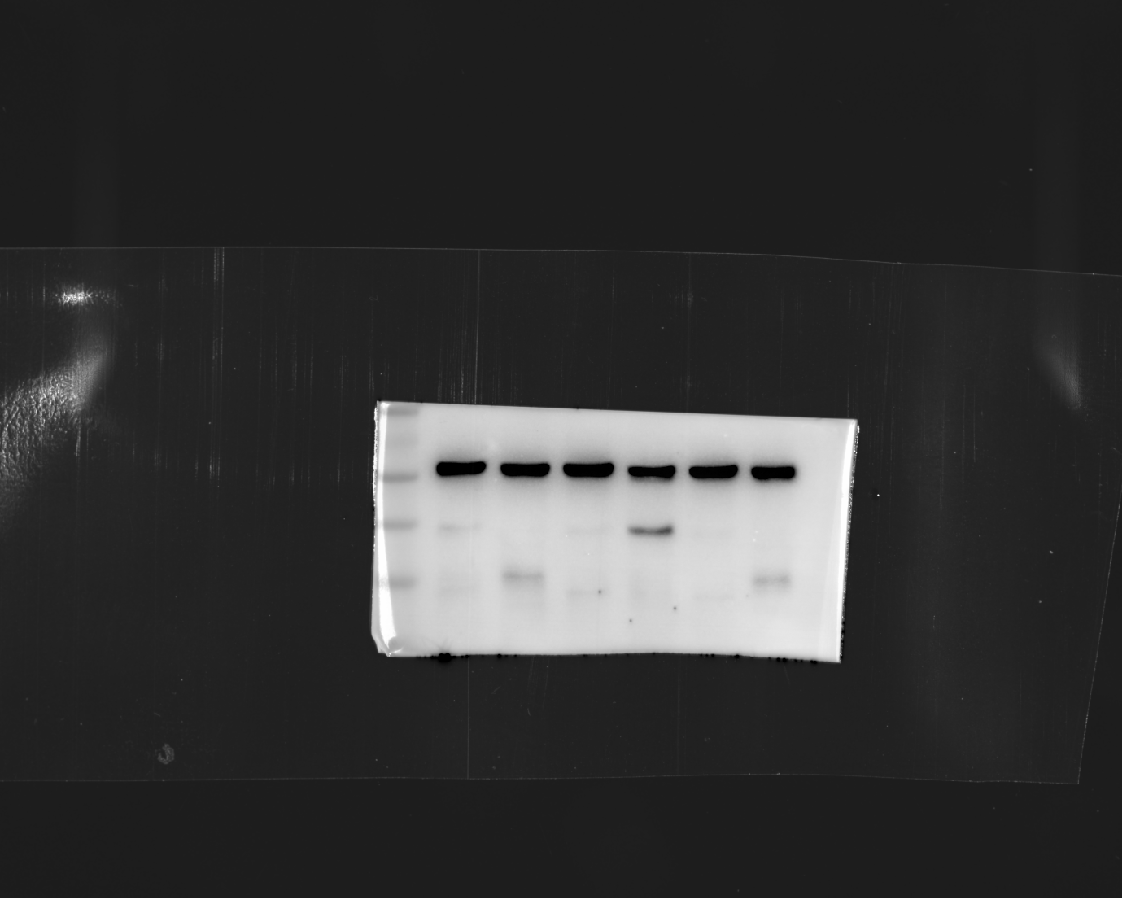


70 kDa

Lamin B1

**Supplementary Fig. 6** Full western blotting images for nuclear phosphor-JNK, total-JNK and lamin B1 band densities. Six middle panels indicate each experimental group at different time points (the first three panels represent control, no-filter and filter groups of day 7, respectively and the last three panels represent control, no-filter and filter groups of day 14, respectively).

**Day 7 (50 µg/lane)**

**Day 14 (50 µg/lane)**

Control

No-filter

Filter

Control

No-filter

Filter


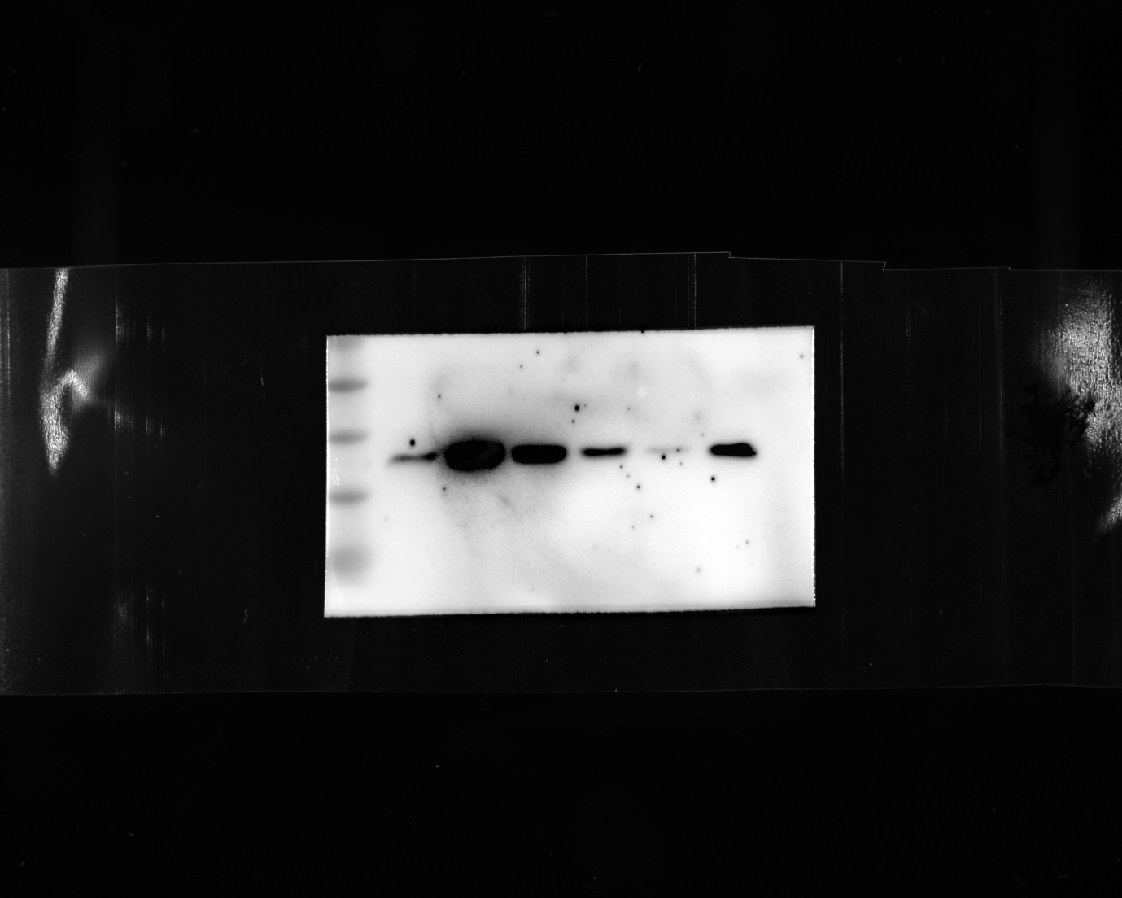


43 kDa

Cytoplasmic phospho-p38


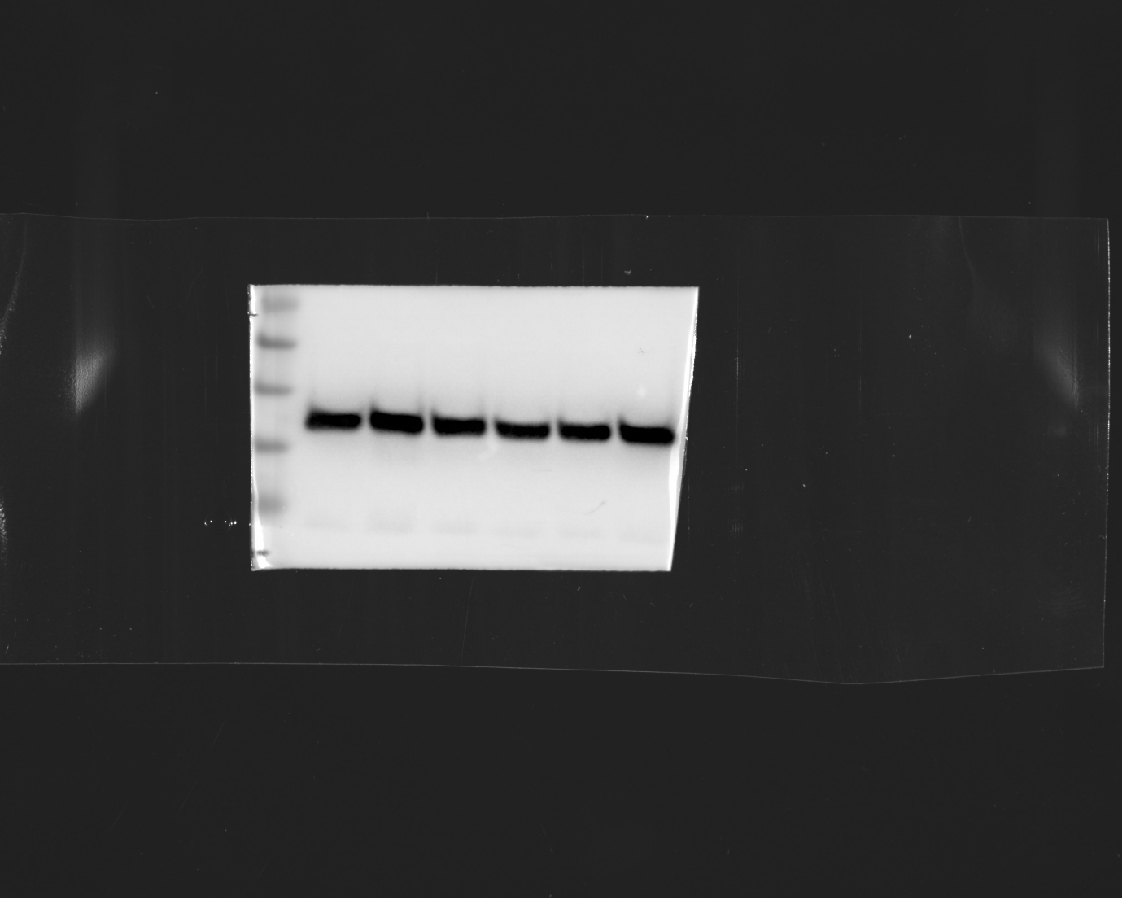


Cytoplasmic total-p38

40 kDa


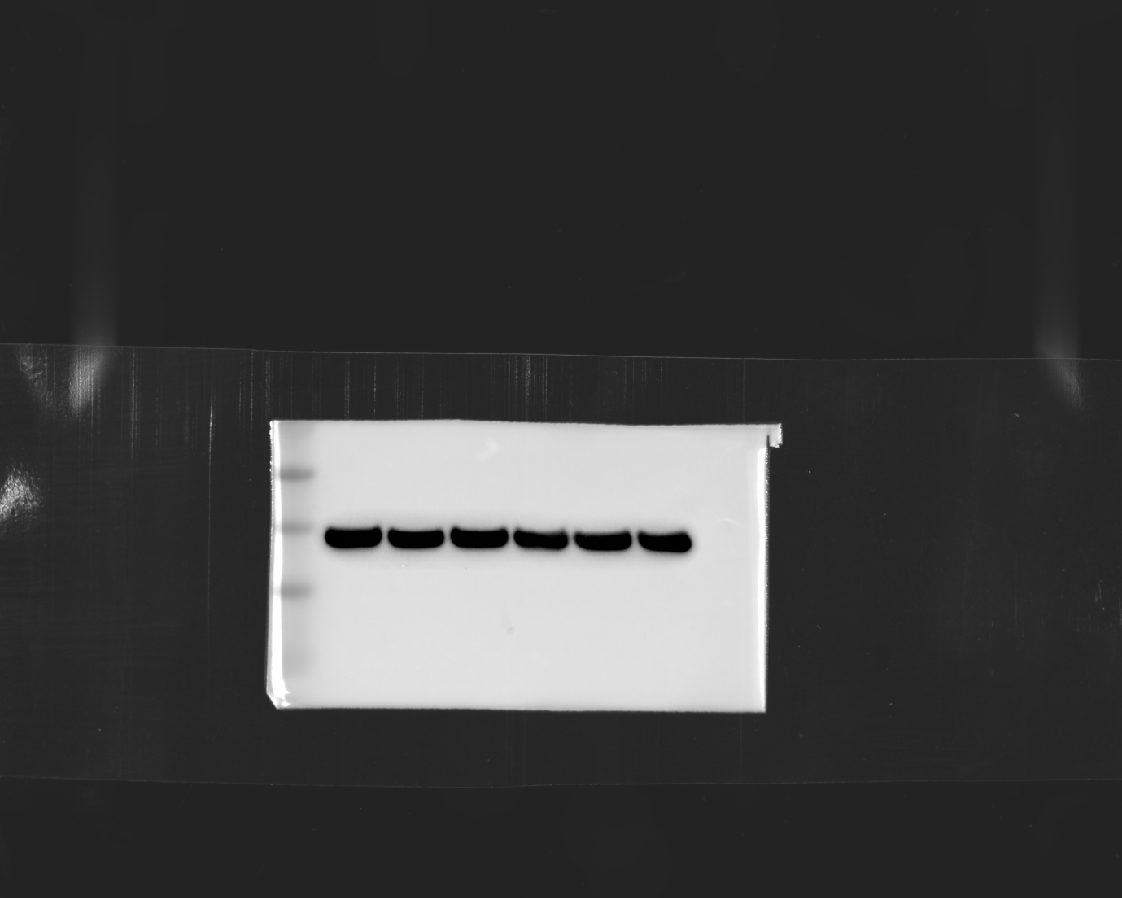


45 kDa

β-actin

**Supplementary Fig. 7** Full western blotting images for cytoplasmic phosphor-p38, total-p38 and β-actin band densities. Six middle panels indicate each experimental group at different time points (the first three panels represent control, no-filter and filter groups of day 7, respectively and the last three panels represent control, no-filter and filter groups of day 14, respectively).

**Day 14 (50 µg/lane)**

Filter

No-filter

Control

Filter

No-filter

Control

**Day 7 (50 µg/lane)**

**
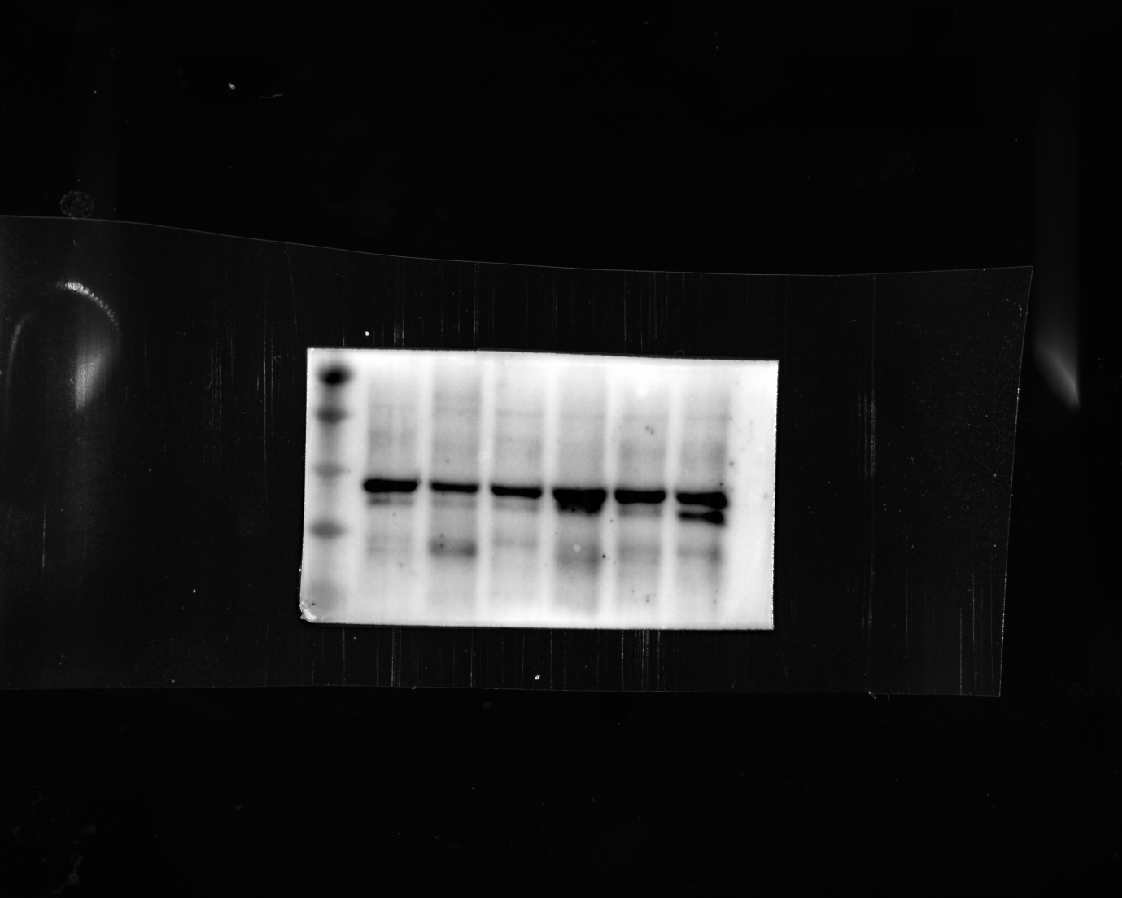
**

43 kDa

Nuclear phospho-p38

**
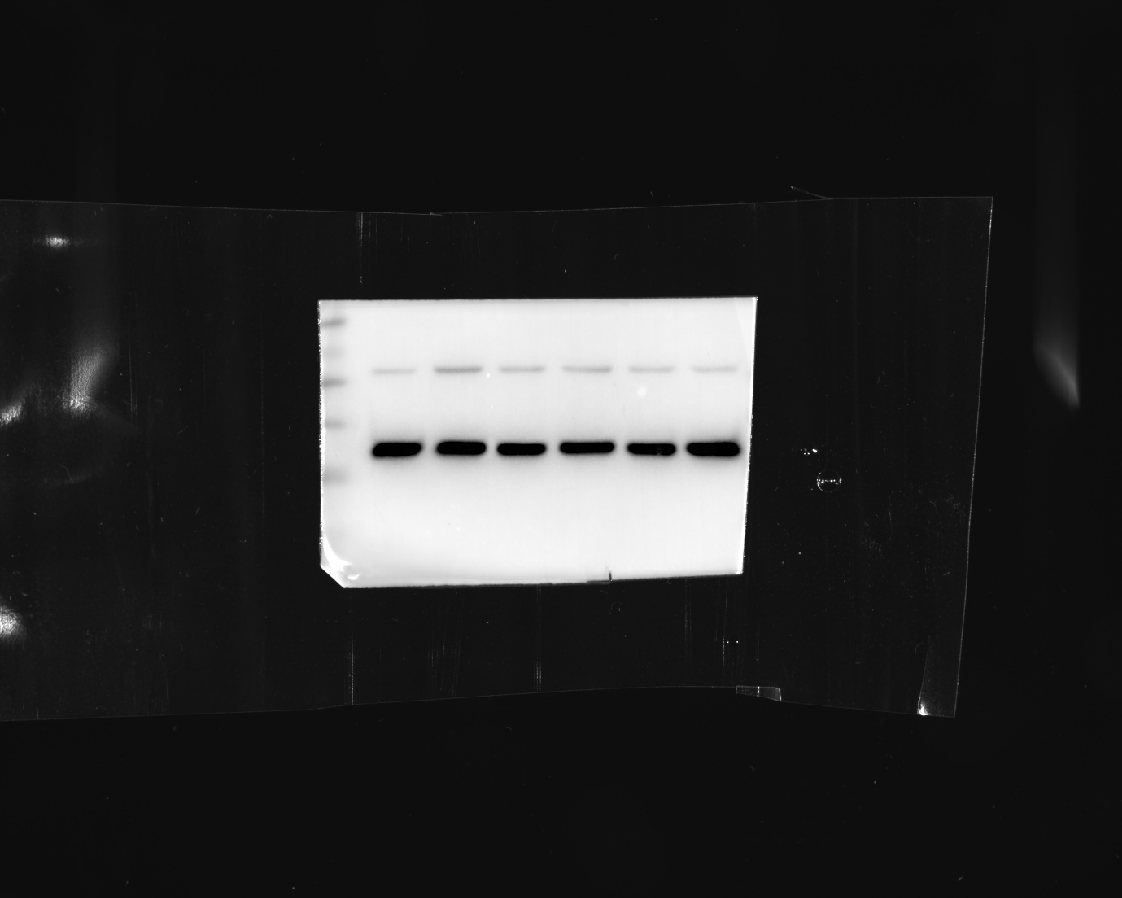
**

40 kDa

Nuclear total-p38

**
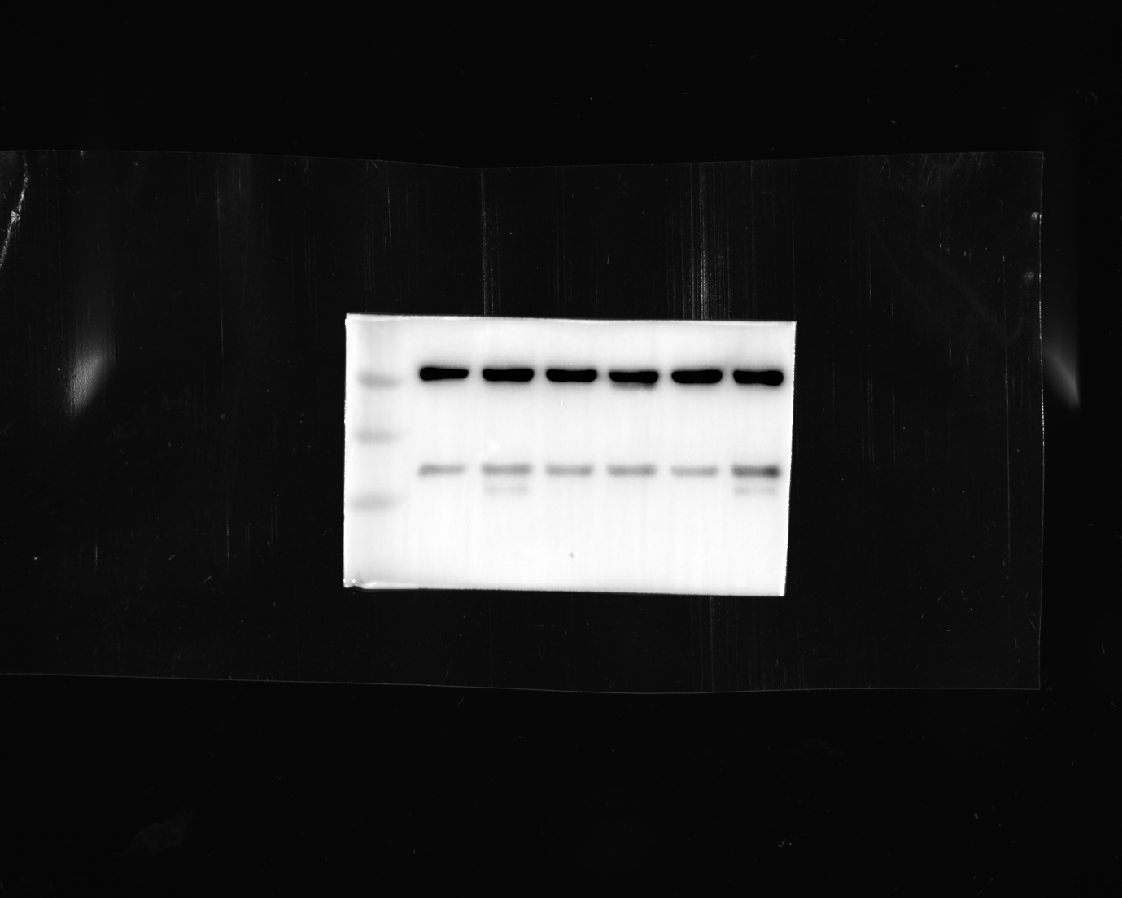
**

70 kDa

Lamin B1

**Supplementary Fig. 8** Full western blotting images for nuclear phosphor-p38, total-p38 and lamin B1 band densities. Six middle panels indicate each experimental group at different time points (the first three panels represent control, no-filter and filter groups of day 7, respectively and the last three panels represent control, no-filter and filter groups of day 14, respectively).
